# High-power continuous-wave optical waveguiding in a silica micro/nanofibre

**DOI:** 10.1038/s41377-023-01109-2

**Published:** 2023-04-07

**Authors:** Jianbin Zhang, Yi Kang, Xin Guo, Yuhang Li, Keying Liu, Yu Xie, Hao Wu, Dawei Cai, Jue Gong, Zhangxing Shi, Yingying Jin, Pan Wang, Wei Fang, Lei Zhang, Limin Tong

**Affiliations:** 1 Interdisciplinary Center for Quantum Information, State Key Laboratory of Modern Optical Instrumentation, College of Optical Science and Engineering, Zhejiang University, 310027 Hangzhou, China; 2 Intelligent Optics & Photonics Research Center, Jiaxing Institute of Zhejiang University, 314000 Jiaxing, China; 3 State Key Laboratory of Precision Measurement Technology and Instruments, Department of Precision Instrument, Tsinghua University, 100084 Beijing, China; 4 Research Center for Intelligent Sensing, Zhejiang Lab, 311121 Hangzhou, China; 5 Collaborative Innovation Center of Extreme Optics, Shanxi University, 030006 Taiyuan, China

**Keywords:** Fibre optics and optical communications, Sub-wavelength optics, Nonlinear optics

## Abstract

As miniature fibre-optic platforms, micro/nanofibres (MNFs) taper-drawn from silica fibres have been widely studied for applications from optical sensing, nonlinear optics to optomechanics and atom optics. While continuous-wave (CW) optical waveguiding is frequently adopted, so far almost all MNFs are operated in low-power region (e.g., <0.1 W). Here, we demonstrate high-power low-loss CW optical waveguiding in MNFs around 1550-nm wavelength. We show that a pristine MNF, even with a diameter down to 410 nm, can waveguide an optical power higher than 10 W, which is about 30 times higher than demonstrated previously. Also, we predict an optical damage threshold of 70 W. In high-power CW waveguiding MNFs, we demonstrate high-speed optomechanical driving of microparticles in air, and second harmonic generation efficiency higher than those pumped by short pulses. Our results may pave a way towards high-power MNF optics, for both scientific research and technological applications.

## Abstract (web summary)

We demonstrate CW optical waveguiding in a silica MNF with power up to 13 W, making it possible for high-speed optomechanical driving of microparticles, and efficient second/third harmonic generation.

## Graphical abstract

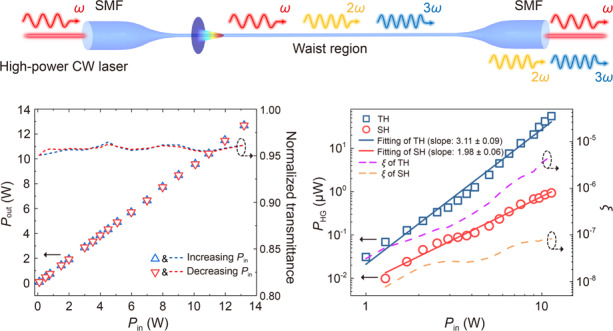

## Introduction

In recent years, optical micro/nanofibre (MNF) taper-drawn from a standard silica fibre has been emerging as a miniature fibre-optic platform with high compactness and great versatilities^1–3^. Benefitting from their intriguing merits including low waveguiding loss, strong evanescent field, tight optical confinement, large engineerable waveguide dispersion, and excellent compatibility with standard optical fibres^2–5^, these MNFs have found wide applications ranging from nonlinear optics^6–10^, optical sensors^11^, atom optics^12–14^ to fibre lasers^15^, and optomechanics^16–18^. Generally, increasing the optical power of the waveguiding modes is the most effective approach to enhance light-matter interaction, and explore new opportunities for both scientific research and technological applications. However, the highest continuous-wave (CW) waveguiding power in a MNF reported so far is ∼0.4 W (ref. ^19^), with most of the rest working below 0.1 W in CW or averaged power.

On the other hand, higher-power CW optical waveguiding in micro/nanowaveguides is highly desired in optomechanics^18,20^, frequency comb generation^21^, nonlinear frequency conversion (harmonic generation, supercontinuum, Brillouin scattering)^6–10,22^, distributed gas sensing^23^, biophotonics^24^, laser source^15,25^, and optical amplification^26^. However, in most of the above-mentioned applications, the waveguiding power is strictly limited by the loss-induced issues, including absorption-induced heating effects^20,27^ or scattering-induced degradation of signal-to-noise ratio^28,29^. In contrast, benefitting from its ultralow optical material absorption (e.g., <10^−6^ in imaginary part of complex refractive index for silica within visible and near-infrared spectral ranges^30^) and extremely low surface roughness (e.g., root-mean-square value of 0.1-nm level^1–3^), a silica MNF can guide light with waveguiding loss well below 0.005 dB cm^−1^ (ref. ^19^), far beyond the reach of all other kinds of micro/nanowaveguides^29^. Also, light guided in a standard single-mode optical fibre with relatively large mode size (e.g., 10-µm level) can be squeezed into a MNF (e.g., sub-1-µm level) almost losslessly (e.g., coupling efficiency >99%)^31,32^ through an adiabatic taper drawn from the same fibre^33–35^. The ultralow waveguiding loss, as well as the negligible coupling loss of a MNF, indicates a great opportunity for its high-power (e.g., >1 W) application.

Here we demonstrate high-power CW optical waveguiding in an optical MNF around 1550-nm wavelength with power up to 13 W, corresponding to a maximum power density of 23 W µm^−2^. We show that, a pristine MNF exhibits an optical transmittance of ∼95% when waveguiding 13-W CW light, even with a diameter down to 410 nm (i.e., ∼*λ*/3.8). By measuring the power-dependent heating effect, we extrapolate an optical damage threshold of ∼70 W (maximum power density of 124 W µm^−2^) in the MNF. In high-power waveguiding MNFs, we realize high-speed optomechanical driving of microparticles, and CW nonlinear frequency conversion with efficiency of 8.2 × 10^−8^ and 4.9 × 10^−6^ for second harmonic generation (SHG) and third harmonic generation (THG), respectively.

## Results

### High-power CW optical waveguiding characteristics

**Fig. 1:**
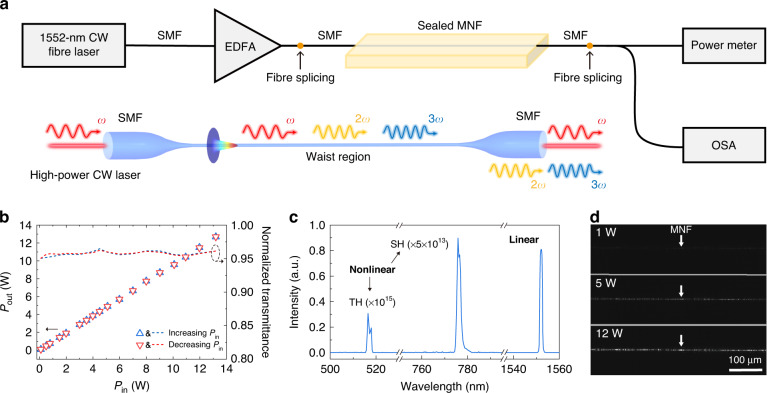
High-power CW optical waveguiding in MNFs around 1550-nm wavelength. **a** Schematic diagram of experimental setup and high-power CW optical waveguiding in a MNF. SMF, single-mode fibre. EDFA, erbium-doped fibre amplifier. OSA, optical spectrum analyzer. **b** Measured optical transmittance of a 1.1-μm-diameter, 2-cm-length MNF with a CW waveguided power from 0 to 13 W. **c** Output spectrum of the MNF with a waveguided power of 12 W. TH, third harmonic, SH, second harmonic. **d** Optical microscope images showing surface scattering in the same 1.1-μm-diameter MNF with CW waveguided power of 1 W, 5 W, and 12 W, respectively. The exposure time is 3 ms in capturing all the three images

In our experiment, high-quality MNFs were fabricated by taper drawing standard single-mode silica fibres (Corning, SMF-28e) via a traveling-stage taper-drawing scheme in a Class 1000 cleanroom^31,36^, with excellent diameter uniformity and surface smoothness (see “Materials and methods” for details). Owing to their adiabatically tapering profile^32–34^, measured optical transmittance of MNFs is routinely larger than 95% (Supplementary Note 1). To investigate the high-power optical waveguiding properties, an as-fabricated MNF was sealed and suspended inside an airtight box filled with high-purity nitrogen gas to keep its pristine surface isolated from possible contamination (Fig. 1a). The CW input light comes from a 1552-nm-wavelength CW fibre laser amplified by a low-noise erbium-doped fibre amplifier (EDFA, Connet, MFAS-Er-C-B-15), which can offer a CW output up to 13 W around 1550-nm wavelength. The input end of the MNF (i.e., the standard single-mode fibre) was fusion spliced to the output fibre of the EDFA with a splicing loss lower than 0.05 dB, and the output of the MNF was measured by a thermopile power meter (Physoe, 0912195) and an optical spectrum analyzer (Yokogawa, AQ6370D). Considering possible hysteretic response of the test system due to the thermal effect, we scanned the input power back and forth from minimum to maximum. As shown in Fig. 1b, the measured output *P*_out_ changes quite linearly with the input power *P*_in_ without observable hysteretic effect, and the MNF maintains a transmittance >95% with waveguided power up to 13 W (corresponding to a maximum power density of 23 W µm^−2^, see Supplementary Note 2), which is more than 30 times higher than the highest power reported before^19^. It is worth mentioning that, the high optical transmittance (∼95%) remains in a MNF with diameter down to 410 nm (i.e., ∼*λ*/3.8, see Supplementary Note 3). The possibility of waveguiding such a high optical power in the MNF can be attributed to the ultralow absorption of silica fibre (used as the preform), high precision in the fibre-pulling process and high cleanliness of both fibre-pulling and testing environment. The long-term stability of high-power operation is also investigated for a 1.2-μm-diameter MNF, showing that the MNF can handle a CW power >10 W without observable degradation with accumulated operation time of more than 10 hours in a 2-month test (Supplementary Note 4). Typical transmission spectrum shows that (Fig. 1c), at the output of a high-power CW waveguiding MNF, besides the linearly transmitted light around 1550 nm, there are very weak nonlinear signals, e.g., second harmonic (SH) and third harmonic (TH) signals, with intensities of more than 13 orders of magnitude lower (measured by a spectrometer, Ocean optics, USB2000+). Surface scattering of a high-power waveguiding MNF is investigated using a short-wave infrared camera (Goldeye, G-033 TECless), showing the increase of scattering intensity with the waveguided power, without predominant single scattering spots (Fig. 1d). The normally distributed scattering intensity (Supplementary Note 5) indicates excellent diameter uniformity and surface smoothness of the MNF. In addition, we find that, the surface field (i.e., waveguided evanescent field on the fibre surface) of a high-power waveguiding MNF can enable a self-cleaning effect: when the fibre surface is contaminated by surface adsorption (e.g., dust from open air), the high-power surface field can clean off the adsorbate and recover the optical transmittance to a certain degree (Supplementary Note 6 and Supplementary video S1).

### Optical damage threshold

**Fig. 2:**
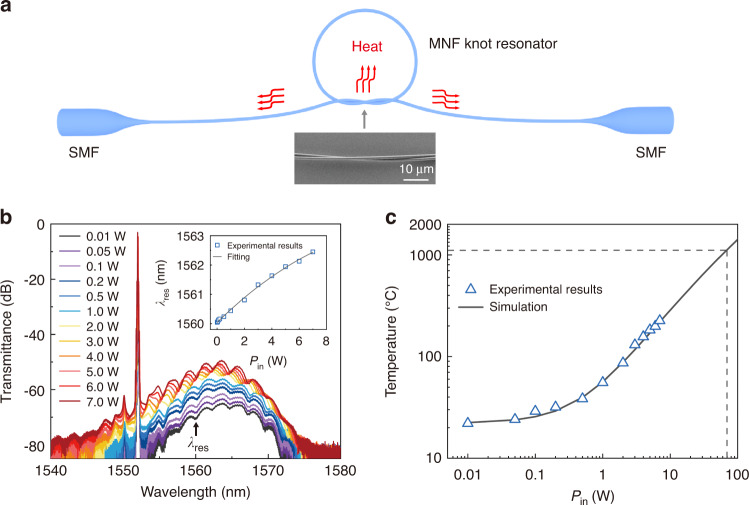
Optical damage threshold of a MNF around 1550-nm wavelength. **a** Schematic diagram of a silica MNF knot resonator for measuring temperature rise in the MNF. Inset, scanning electron microscope image of the intertwisted area of the knot employed, which is ∼230 µm in diameter and is assembled using a 1.2-µm-diameter MNF. **b** Transmission spectra of the knot with waveguided power increasing from 0.01 W to 7.0 W. Inset, power-dependent spectral shift of the resonance peak at *λ*_res_ marked by an arrow. **c** Measured (blue triangle) and calculated (black line) power-dependent temperature of the MNF, with an extrapolated damage threshold of ~70 W at a temperature of 1100 °C

As the MNF can transmit a CW power higher than 10 W without detectable degradation (Supplementary Note 4, in nitrogen gas), the optical damage threshold should be much higher than the waveguiding power available from a current single-mode EDFA (i.e., <15 W). To estimate the optical damage threshold, we measure the power-dependent temperature rise of the MNF within the available power range, and extrapolate the damage threshold based on a thermal dissipation model. To ensure the availability and accuracy of the temperature measurement, we assembled a 1.2-µm-diameter MNF into a 230-µm-diameter knot resonator^37^, measured the power-dependent spectral shift of the resonant peak (Fig. 2a, b), and obtained the temperature rise of the MNF after calibration (Supplementary Note 7). It is worth mentioning that, power-dependent temperature rise in waveguiding MNFs in low-power region (at the milliwatt level) has been previously measured with high-quality cavity^38^ or heterodyne detection^39^. Here for measuring temperature rise in high-power region, we used such a knot resonator with a relatively low quality (quality factor of ~1800 and finesse of ~2.7) to avoid excessive power (and thus heat) accumulation. The transmission spectra clearly show that, with increasing power, the resonant peaks of the MNF knot exhibit a monotonous red-shift (inset, Fig. 2b), which is used to retrieve the temperature of the MNF based on the thermo-optic and thermal expansion effects of the silica MNF knot resonator^40^. With the power-dependent temperature rise and a nearly constant optical absorption coefficient of the silica MNF over a broad temperature range (Supplementary Note 8), the temperature of a MNF waveguiding a higher power can be extrapolated using a thermal dissipation model calibrated with available experimental data (Fig. 2c, see also “Materials and methods” section and Supplementary Note 9). Considering the typical upper limit of operation temperature (~1100 °C) of a silica fibre (i.e., annealing temperature of the silica^41,42^), the optical damage threshold is estimated to be ~70 W (Fig. 2c), which is 5 times higher than the highest available power (~13 W) used in this work.

However, the power density of the estimated damage threshold (e.g., ~140 W µm^−2^ in a 1.2-µm-diameter MNF) is still much lower than those reported in pure silica (e.g., 5 kW µm^−2^ for laser-induced stimulated Brillouin scattering and 54 kW µm^−2^ for self-focusing effects^43,44^, 2 typical damage mechanisms in silica glass), which can be attributed to much higher defect absorption in the MNF. Compared with the bulk silica, the MNF fabricated by a flame-heated taper drawing process at high temperature leaves much higher population of defects including surface defects (e.g., oxygen deficient center and oxygen dangling bonds) and water molecules (OH^−^), as have been confirmed experimentally in photoluminescence and transmission spectra^45–47^ (Supplementary Notes 10 and 11). Nonetheless, higher optical transmittance (e.g., >99%) can be obtained in a MNF^32^, and surface defects such as oxygen dangling bonds and peroxy radical will annihilate above 600 °C, which may reduce optical absorption at higher power^48^. Also, the thermal dissipation via thermal convection in air has not been included. Therefore, the real optical damage threshold could be much higher than that estimated here.

### High-power CW optomechanical manipulation

**Fig. 3:**
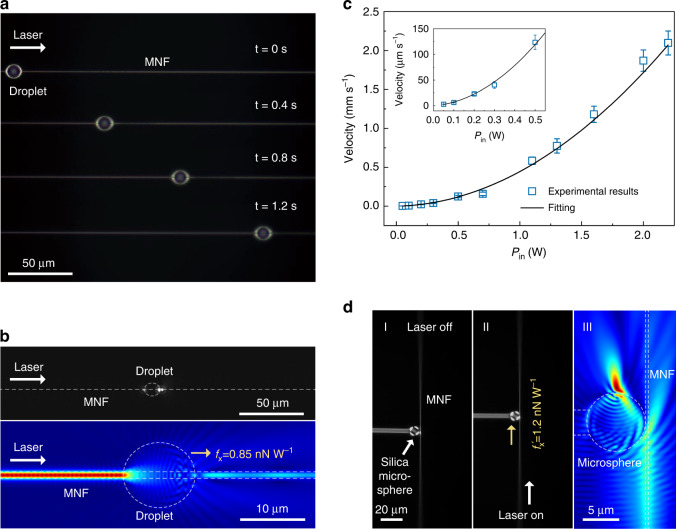
High-power CW optomechanical manipulation using MNFs waveguiding a 1552-nm-wavelength light. **a** Time-sequential optical microscope images of driving an oil droplet (11 μm × 10 μm ellipsoid) along a 1-μm-diameter MNF at 0.4-s intervals. A 0.7-W-power light is waveguided along the MNF from left to right. **b** Optical microscope image (upper) and field intensity calculation (bottom) of the fundamental waveguiding mode in a 1-μm-diameter MNF scattered by an oil droplet wrapped around. **c** Measured (blue squares) and fitted (black line) power-dependent velocity of the oil droplet driven by the waveguiding light in the MNF. Inset, a close-up of the droplet velocity with waveguided power below 0.5 W. **d** Optical microscope image (I, II) and field intensity calculation (III) of optomechanical manipulation of an 8-μm-diameter silica microsphere via an evanescent field of a 410-nm-diameter MNF. The gap between the microsphere and the MNF is ~0.1 μm. The waveguided power is 4 W. The white dash lines in b and d indicate the geometric profiles of the MNF, the droplet and the silica microsphere, respectively

The evanescent field waveguided along the MNF offers a flexible and precise platform for optomechanically manipulating (e.g., trapping or propelling) microparticles^16–18^. Previously, with relatively low waveguided power (<0.1 W), almost all manipulations were performed in liquid environment to alleviate influence effects such as adhesion surface force^49^ and gravity. Here, with much higher optical power and thus much larger optical force (Supplementary Note 12), it is possible to drive microparticles in air or vacuum with higher speed. Figure 3a shows optomechanically driving a silicone oil droplet (Xiameter, PMX-200) using a high-power CW waveguiding MNF. The ellipsoid-shaped oil droplet (11 μm in major axis and 10 μm in minor axis) is wrapped around a 1-μm-diameter MNF in air. A 1552-nm-wavelength CW light, used as the propelling light, is coupled into and waveguided along the MNF from left to right to drive the droplet (Fig. 3b). The droplet keeps motionless until the light power is increased beyond 0.02 W, corresponding to an adhesion force of ∼17 pN (calculated from an optical force of 0.85 nN W^−1^, see Supplementary Note 12 and Supplementary video S2). When the light power exceeds 0.04 W, the oil droplet can be propelled to slide instantly along the MNF (Supplementary video S2). The measured power-dependent droplet velocity *v*_o_ (e.g., *v*_o_ = 2.1 mm s^−1^ at a waveguided power of 2.2 W, see Fig. 3c and Supplementary video S3) shows that the droplet can be driven more than 10 times faster than those reported in previous MNF-based optomechanics systems^16,18,50^. The nonlinear power-velocity behavior (with a fitting function *v*_o_ ≈ *A* × *P*_in_^2^ + *B* × *P*_in_, *A* = 4.1×10^−4^ m s^−1^ W^−2^, *B* = 3.0×10^−5^ m s^−1^ W^−1^) can be attributed to a photothermal effect, in which the viscosity of the silicone oil decreases due to the temperature rising with the increasing power, resulting in a nonlinear dependence of the velocity on waveguided power^51^. The MNF-guided high-power evanescent field can also offer non-contact optomechanical manipulation of relatively large objects in air. When an 8-μm-diameter silica microsphere fabricated on the tip of a 3.2-μm-diameter fibre taper was moved into the near field of a 410-nm-diameter MNF waveguiding a 4-W-power 1552-nm-wavelength CW light (axial force *F*_x_^′^=4.8 nN, see Supplementary Note 13), the microsphere shows an evident displacement (Supplementary video S4). As shown in Fig. 3d, with a microsphere-to-MNF gap of 0.1 μm, the microsphere manifests an instant displacement of ~14 μm upon the onset of a CW 4-W 1552-nm waveguiding light (Supplementary video S4), as an optomechanical response to the waveguided evanescent field. The optomechanical response of the microsphere can be precisely tuned by the waveguided power, with measured displacement agreeing very well with that obtained by theoretical calculation (Supplementary Note 14). Since optomechanical manipulation in non-liquid environment is critical to optical precision metrology^52^ and quantum optics^53^, the high-power MNF manipulation of microscale objects in air may pave a step toward a fibre-based platform for high-speed optomechanical manipulation in non-liquid atmosphere or vacuum.

### High-power CW nonlinear harmonic generation

Owing to the tight optical confinement, surface field enhancement and diameter-dependent dispersion of a waveguiding MNF^4^, MNF-based nonlinear optical effects have been attracting increasing interests in recent years^6–10,54,55^. Due to the low optical nonlinearity of silica, usually these effects were generated by short pulses (Supplementary Table S1), although CW nonlinear effects are also desired in standard optical fibres and MNFs^8,10,56^. Here we investigate the possibility of the THG and SHG in MNFs with high-power CW waveguiding. To optimize the conversion efficiency of THG, we choose the MNF diameter to achieve phase matching and maximum mode overlapping between the fundamental and the TH modes (Supplementary Note 15). Meanwhile, unlike in standard glass fibre that the SH is neglectable due to the centrosymmetry of the silica matrix, the large surface-to-volume ratio of the MNF makes it possible to generate evident SH signal on its surface where the symmetry is broken^57^. Thus, the high-power CW waveguiding MNF can also realize evident SHG that was previously possible under pulsed excitation. Coincidentally, for the 1550-nm-wavelength fundamental mode, phase-matching condition for HE_12_(3*ω*) and HE_21_(2*ω*) modes gives approximately the same MNF diameter (Supplementary Note 16).

**Fig. 4:**
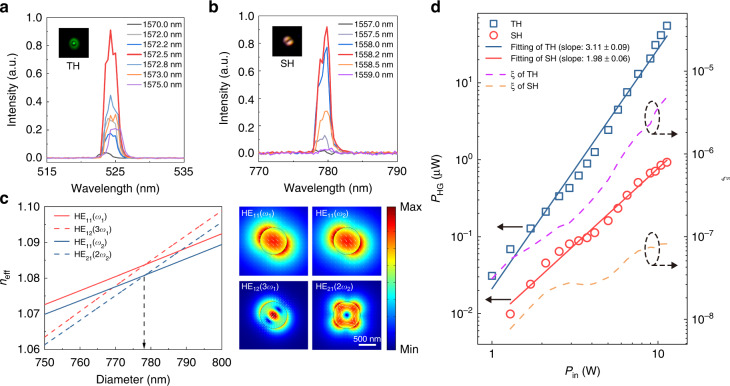
Phase-matched CW harmonic generation in MNFs. **a**, **b** Experimentally measured THG (**a**) and SHG (**b**) spectra in a 779-nm-diameter, 7-cm-length MNF waveguiding a 5-W-power light. To search the optimal phase matching condition, the wavelength of the light is scanned from 1570 nm (1557 nm) to 1575 nm (1559 nm) for THG (SHG). Insets, optical microscope images of output spots of the TH and SH at the endface of the standard fibre connected with the MNF. **c** Calculated effective refractive indices (*n*_eff_) of the fundamental (HE_11_) and high-order (HE_12_ and HE_21_) modes showing phase-matching condition at two intersection points with nearly the same diameter of ∼779 nm at wavelengths of *λ*_1_ = 1572.5 nm for THG (blue lines) and *λ*_2_ = 1558.2 nm for SHG (red lines), respectively. Inset, calculated field intensity profiles of phase-matched waveguiding modes for THG and SHG, respectively. **d** Dependence of the output power (*P*_HG_) of THG/SHG on the waveguided power (*P*_in_). The fitting slopes for the THG and SHG are 3.11 ± 0.09 and 1.98 ± 0.06, respectively. *ξ*, conversion efficiency of harmonic generation

Experimentally, a MNF with diameter of 779 nm and length of 7 cm was fabricated with high-precision diameter control using direct mode cut-off feedback^36^ (Supplementary Note 17) and was isolated in an acrylic chamber (Supplementary Note 18). Using a high-wavelength-accuracy tunable laser (Santec, TSL-710) as the seed source for fine wavelength tuning (Supplementary Note 19), the perfect intermodal phase matching of THG (HE_11_(*ω*_1_) and HE_12_(3*ω*_1_) modes) and SHG (HE_11_(*ω*_2_) and HE_21_(2*ω*_2_) modes) is realized with *ω*_1_ = 190.8 THz (*λ*_1_ = 1572.5 nm) and *ω*_2_ = 192.5 THz (*λ*_2_ = 1558.2 nm) for the 779-nm-diameter MNF (Fig. 4a, b), agreeing very well with theoretical calculations (Fig. 4c). At the phase matching condition (*λ*_1_ = 1572.5 nm, *λ*_2_ = 1558.2 nm), the TH/SH intensity is maximized and much higher than those under phase-mismatching condition in Fig. 1c (e.g., 6 or 10 orders of magnitude higher in SH or TH intensity). At the output fibre endface, the field patterns of LP_02_ mode at 524.2-nm wavelength and LP_11_ mode at 779.1-nm wavelength (insets, Fig. 4a, b) correspond well to the phase-matching modes of HE_12_(3*ω*) mode for THG and HE_21_(2*ω*) mode for SHG in the MNF (inset, Fig. 4c), respectively. Also, by comparing the output spectra of the MNF with a standard glass fibre under high-power CW waveguiding, we confirm that the TH and SH signals come solely from the MNF (Supplementary Note 20).

To investigate the frequency conversion efficiency in the high-power CW waveguiding MNF, the dependence of THG and SHG output on the waveguided power is investigated under phase matching condition (i.e., *λ*_1_ = 1572.5 nm for THG, and *λ*_2_ = 1558.2 nm for SHG) with waveguided power up to 11.3 W (Fig. 4d). The approximately linear log-log dependence gives a fitting slope of 3.11 ± 0.09 for the THG and 1.98 ± 0.06 for the SHG, agreeing very well with the cubic and quadratic dependences for the THG and SHG, respectively. At a waveguided power of 11.3 W, the frequency conversion efficiency is 8.2 × 10^−8^ for the SHG, which is higher than those reported previously using short pulses (e.g., 2.0 × 10^−9^ for the SHG with 400-ps pulsed excitation^58^, see also Supplementary Table S1); the THG efficiency is 4.9 × 10^−6^, falling in the range of typical results reported previously using short pulses (see Supplementary Table S1). The extraordinary CW conversion efficiency comes from combined factors of high waveguiding power, perfectly matched phase and relatively large interaction length. In addition, as the power we used here is lower than the damage threshold (70 W), the nonlinear frequency conversion efficiency could be even higher when higher CW waveguiding power is available (e.g., 1.9 × 10^−4^ for THG and 5.1 × 10^−7^ for SHG, extrapolated from Fig. 4d with *P*_in_ = 70 W).

## Discussion

We have demonstrated high-power CW optical waveguiding in silica MNFs around 1550-nm wavelength. We show that a pristine MNF can safely waveguide a CW power higher than 10 W with diameter down to 410 nm, while maintaining an optical transmittance higher than 95% with excellent long-term stability. Based on absorption-induced thermal effect, we predict an optical damage threshold of 70 W in CW power. With high-power CW waveguiding MNFs, we have also demonstrated high-speed optical driving of microparticles in air, and intermodal-phase-matched second harmonic generation with efficiency higher than those pumped by short pulses. As CW waveguiding is desired in a variety of MNF-based applications noted above, we foresee that our results may extend MNF optics into high-power region, and open up new opportunities for MNF-based technology ranging from fibre laser, nonlinear conversion, optomechanics to biophotonics and atom optics.

## Materials and methods

### Fabrication of MNFs

The MNF was taper drawn from a standard single-mode fibre (Corning, SMF-28e) via a high-precision traveling-stage taper-drawing system. A standard single-mode fibre was fixed on fibre clamps and preheated for ~100 s by stationary hydrogen flame to be softened. Next, the optical fibre was firstly pulled on both ends and then the center of fibre moved to and fro to mimic the flame brushing process. During the pulling process, the real-time transmission of the MNF was measured in-situ, and the MNF diameter was precisely controlled using high-order-mode cut-off feedback^36^. Finally, a high-quality MNF was obtained and sealed inside an acrylic chamber. The whole fabrication process was operated in a Class 1000 cleanroom.

### Calculation of the MNF temperature evolution

From thermal dynamics aspects, the dominant heat dissipation channel is thermal conduction along the MNF. The rest of heat is transferred to the air through thermal conduction, thermal radiation and convection. Our experimental measurement of MNF temperature is modeled with the equations of thermal dissipation including the Fourier law of heat conduction and Stefan-Boltzmann law. Based on the thermal equilibrium between optical absorption induced heat and heat dissipation of the MNF into free space (assume to be vacuum), we calculate the temperature of the MNF at a steady state using a finite element method (Comsol Multiphysics). The waist of the MNF is embedded in the air and the thermal power for heating the MNF *P*_heat_ that is generated by absorbing waveguiding power *P*_in_ can be expressed as *P*_heat_ = *P*_in_ × *α*_abs_, where *α*_abs_ is the overall absorption coefficient of the MNF. Based on the thermal dissipation simulation of the MNF and experimentally measured power-dependent temperature results, we obtain the value of *α*_abs_ of ~1% (Supplementary Note 9). Finally, the calculated MNF temperature versus waveguided power is obtained to extrapolate the optical damage threshold of the MNF, as shown in Fig. 2c.

## Supplementary information

Supplementary Information

Supplementary Video S1

Supplementary Video S2

Supplementary Video S3

Supplementary Video S4
