# Supplementary material for: High-power continuous-wave optical waveguiding in a silica micro/nanofibre
Source: Light Sci Appl. 2023 Apr 7;12:89. doi: 10.1038/s41377-023-01109-2 (PMC10082085; doi:10.1038/s41377-023-01109-2)
Supplement: Supplementary file 1 — Supplementary Information [file 41377_2023_1109_MOESM1_ESM.docx]

Supplementary Information for

High-Power Continuous-Wave Optical Waveguiding in a Silica Micro/Nanofibre

Jianbin Zhang^1,†^, Yi Kang^1,†^, Xin Guo^1,2,*^, Yuhang Li^3,*^, Keying Liu^1^, Yu Xie^1^, Hao Wu^1^, Dawei Cai^1^, Jue Gong^1^, Zhangxing Shi^1^, Yingying Jin^1^, Pan Wang^1,2^, Wei Fang^1,2^, Lei Zhang^1,4^ and Limin Tong^1,2,5,*^

*^1^Interdisciplinary Center for Quantum Information, State Key Laboratory of Modern Optical Instrumentation, College of Optical Science and Engineering, Zhejiang University, Hangzhou 310027, China*

*^2^Intelligent Optics & Photonics Research Center, Jiaxing Institute of Zhejiang University, Jiaxing 314000, China*

*^3^State Key Laboratory of Precision Measurement Technology and Instruments, Department of Precision Instrument, Tsinghua University, Beijing 100084, China*

*^4^Research Center for Intelligent Sensing, Zhejiang Lab, Hangzhou 311121, China*

*^5^Collaborative Innovation Center of Extreme Optics, Shanxi University, Taiyuan 030006, China*

*^*^Correspondence: Xin Guo (*[*guoxin@zju.edu.cn*](mailto:guoxin@zju.edu.cn)*) or Yuhang Li (*[*liyuhang@tsinghua.edu.cn*](mailto:liyuhang@tsinghua.edu.cn)*) or Limin Tong (*[*phytong@zju.edu.cn*](mailto:phytong@zju.edu.cn)*)*

*^†^These authors contributed equally to this work*

Supplementary Note 1. Optical characterization of the as-fabricated MNFs

Typical optical transmittance during a complete fabrication process of pulling a standard single-mode silica fibre into an optical MNF (down to a diameter of 900 nm and a length of 2 cm) was monitored by a 1550-nm-wavelength CW light (Joinwit, JW3116). As illustrated in Fig. S1a, continuous oscillations occurred at about 7 s, corresponding to weak coupling and interference between the fundamental mode and high-order modes in the fibre until the MNF was thin enough for single-mode operation at about 75 s. The small fluctuations starting from ~95 s came from the minor disturbance of the flame airflow (causing micro-bending loss of the MNF) during the sweeping process. When the pulling process was finished and the MNF was tensioned to straight, the total transmittance of the as-fabricated MNF (including the transition regions at both sides) was higher than 95% around 1550-nm wavelength.

To study the broadband optical waveguiding characteristics of the MNF, a white light (a fibre-coupled tungsten-halogen light, Thorlabs, SLS201L) was launched into one side of the MNF and the output signal was collected from the other side by two spectrometers (Ocean optics, USB2000+ for visible and NIR Quest512 for near-infrared spectral ranges, respectively). The measured broadband (500-1700 nm) transmission spectrum of the MNF showed that, there were three rising edges in the transmission curve, corresponding to cut-off wavelengths of different high-order modes (marked with vertical blue dashed lines and corresponding mode indices) around 630 nm, 810 nm and 1250 nm, respectively (Fig. S1b). The abrupt dip around 1400-nm wavelength originated from the absorption of water molecules (OH^-^).

To intuitively illustrate the profile of the MNFs with transition regions at both sides, we measured the diameter evolution of the tapering regions (Fig. S1c, red circles) with relatively large diameter using an optical microscope (upper insets), and that of the MNF (Fig. S1c, blue circles) with small diameter (e.g., < 5 μm) using a scanning electron microscope (SEM, bottom inset). Figure S1c shows schematic (upper, not to scale) and experimentally measured (bottom) fibre diameter evolution of a biconically drawn fibre-MNF-fibre structure along the fibre length. The MNF in the waist area is about 930 nm in diameter and 9 cm in length. The SEM image (Fig. S1c, bottom inset) reveals an extremely smooth surface and uniform diameter (0.93 ± 0.02 μm) of the MNF.


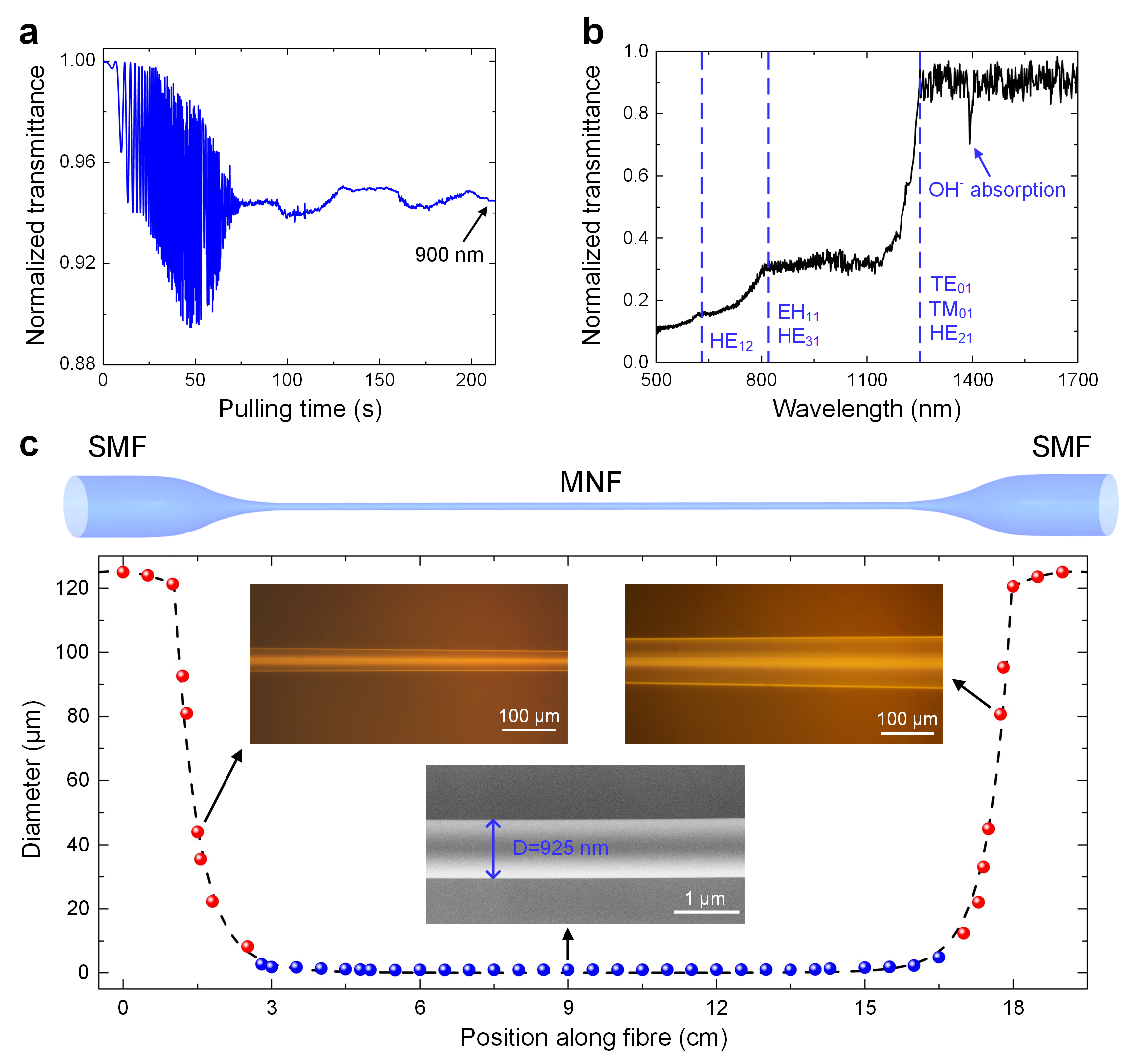


Fig. S1. Optical transmission and [characterization](http://www.baidu.com/link?url=HVSNzWcxiHt_SbJvKFDtB2V18A8WtKoqJUUh47OxivzfsirsVQI_7oreUm6B9jnz3WruRODtnpz8TmjEozyadKB2BEUyAFJ1Edz61zC50jXFxApxkijM3xhgcsaRz6lF) of MNFs. a Typical pulling-time-dependent optical transmittance of a 1550-nm-wavelength light during the fabrication of a MNF with a final diameter of 900 nm and a length of 2 cm. b Broadband optical transmission spectrum of the MNF showing cut-off of different high-order modes (marked with vertical blue dashed lines and corresponding mode indices) and an absorption peak of OH^-^. c Schematic (upper, not to scale) and experimentally measured (bottom) fibre diameter evolution of a biconically drawn fibre-MNF-fibre structure along the fibre length. SMF, single-mode fibre. The MNF in the waist area is about 930 nm in diameter and 9 cm in length. The diameter evolution of the tapering region (red circles) was measured using an optical microscope (upper insets), while that of the MNF (blue circles) was measured using a scanning electron microscope (bottom inset).

Supplementary Note 2. Calculated power density of the optical MNFs


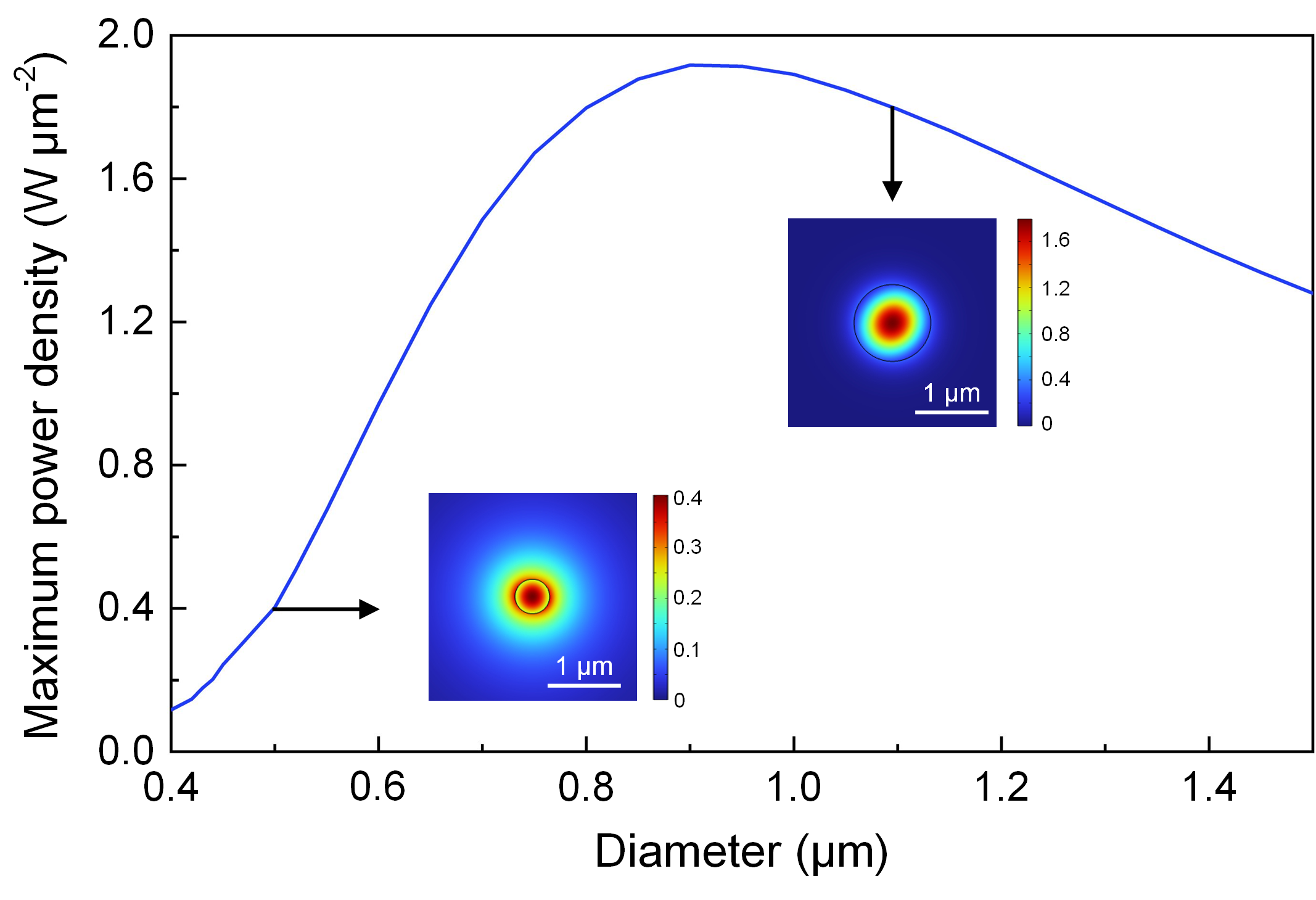


Fig. S2. Calculated diameter-dependent maximum power density in MNFs at 1552-nm wavelength with a waveguided power of 1 W. Insets, cross-sectional power density distribution of silica MNFs with diameters of 0.5 μm and 1.1 μm, respectively. The calculation was performed by finite element method (Comsol Multiphysics). For the 1.1-μm-diameter MNF used in the experiment, the maximum power density is about 23 W µm^-2^ when waveguiding a 13-W CW light.

Supplementary Note 3. High-power CW optical waveguiding in a 410-nm-diameter MNF

In the experiment, we found that, a pristine MNF with a diameter of 410 nm (corresponding to ~*λ*/3.8 at the wavelength *λ* of 1552 nm) maintained an optical transmittance of ~95% with a CW waveguided power of ~13 W in the air, as shown in Fig. S3.


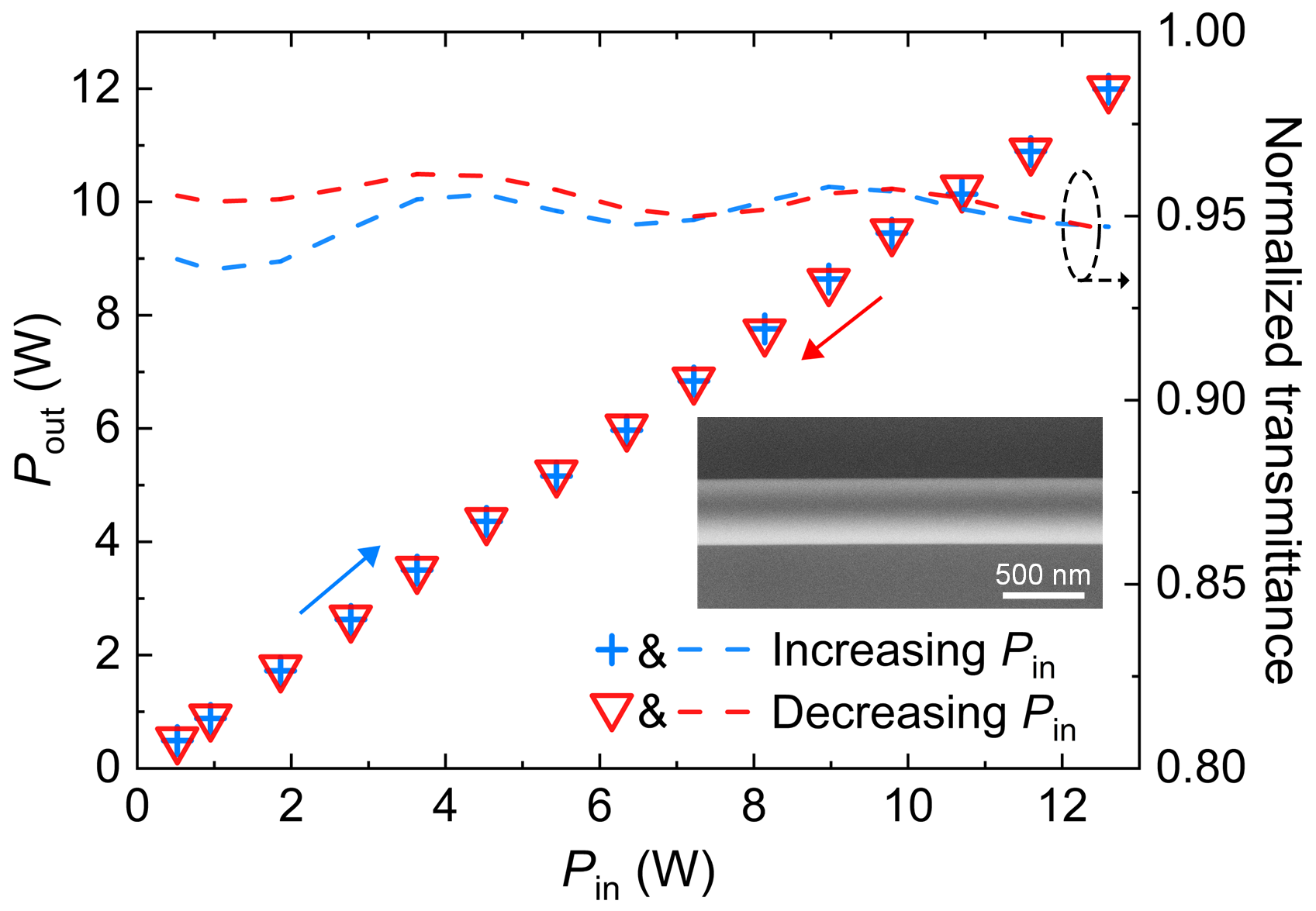


Fig. S3. Measured optical transmission of a 410-nm-diameter 2-cm-length MNF with a 1552-nm-wavelength CW waveguided power from 0 to ~13 W in the air. Inset, a SEM image of the MNF.

Supplementary Note 4. Encapsulation and long-term high-power operation of a MNF

An as-fabricated MNF was transferred to a clean and airtight 3D-printed acrylic box, with the two untapered fibre at both sides fastened on a silicone holder, as shown in Fig. S4a. The airtight box adopted a hollow structure to protect the MNF without any contact, filled with high-pure nitrogen gas to eliminate possible air contamination of the box. The pressure and temperature of the nitrogen gas in the box that contained the MNF were maintained at one atmospheric pressure (~0.1 MPa) and room temperature (~22 ℃), respectively.

To investigate the long-term stability of the high-power waveguiding MNF, we measured 1552-nm-wavelength optical transmission of an encapsulated 1.2-μm-diameter 2-cm-length MNF with a waveguided CW power of 12 W. To protect the EDFA from heating-induced overload, we tested at 12-W power continuously for 20 minutes every day for two months. The results in Fig. S4b showed that, over the whole test period of two months, the optical transmittance of the MNF maintained around 95%, without observable degradation within an accumulated high-power operation time of more than 10 hours.


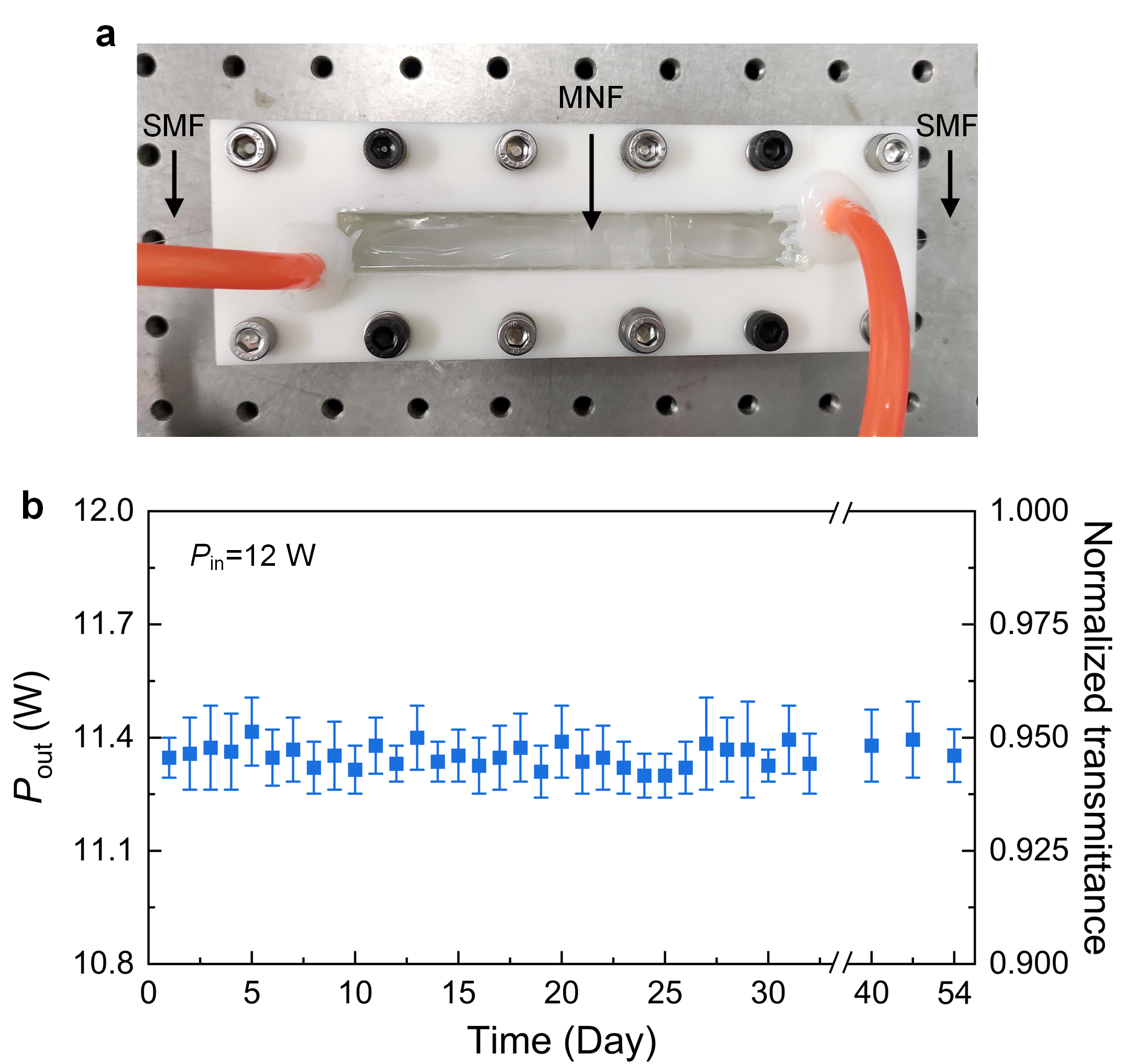


Fig. S4. Encapsulation (a) and long-term optical transmission (b) of a 1.2-μm-diameter 2-cm-length MNF with a waveguided CW power of 12 W.

Supplementary Note 5. Statistical analysis of the surface scattering intensity along the length of a MNF

Optical microscope images of surface scattering along the length of a 1.1-μm-diameter 2-cm-length MNF waveguiding a 5-W-power 1552-nm-wavelength light are presented in Fig. S5a. To investigate the scattering intensity distribution, we performed a statistical analysis of the surface scattering intensity at three different positions (I: 0.5 cm, II: 1.0 cm and III: 1.5 cm away from the left side of the MNF, Fig. S5a), and captured a 570-μm-length segment of the MNF around each position using a short-wave infrared camera (Goldeye, G-033 TECless) with dark background. Within each segment, we implement a statistical analysis by equally dividing it into 190 square intervals along the fibre length, and each interval has an area of 9 μm² that contains 100 pixels with a grayscale value of 0 to 255 for each pixel. Figure S5b gives the statistical results of the grayscale value (a measure of the scattering intensity), showing very similar distribution at the three positions. The normal probability plot confirms that the surface scattering distribution agrees well with normal distribution (Fig. S5c), indicating the high surface quality and diameter uniformity of the as-fabricated MNF, as any obvious structural defect will lead to abruptly large scattering intensity that deviates the normal distribution.


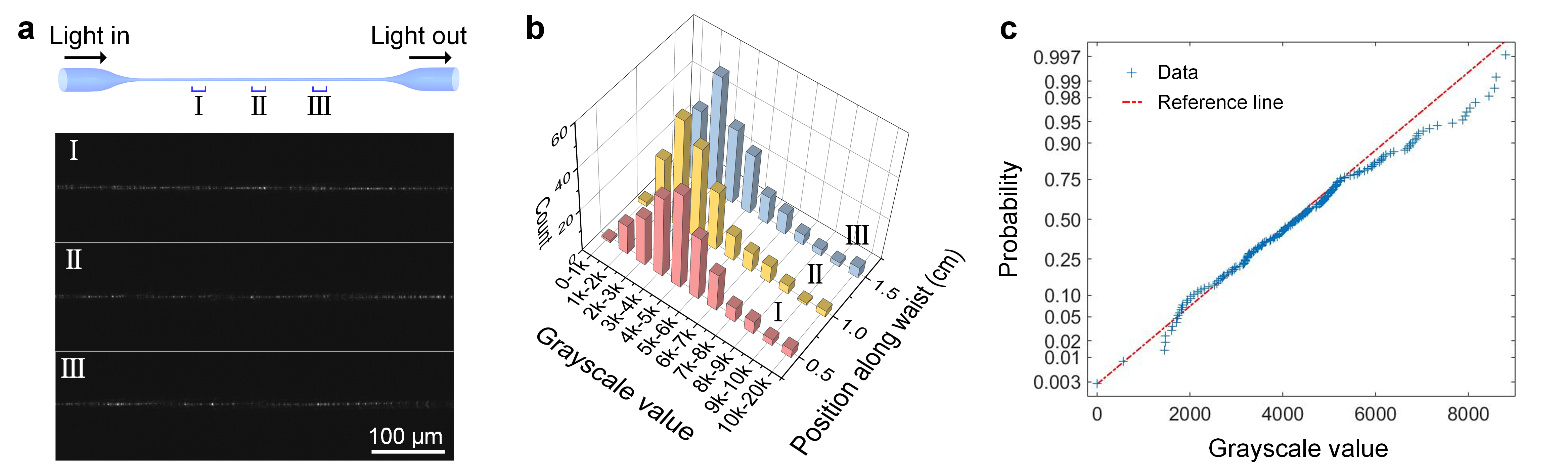


Fig. S5. Statistical analysis of the surface scattering intensity of a MNF. a Schematic (upper) and optical microscope images (bottom) of investigating surface scattering along the length of a 1.1-μm-diameter 2-cm-length MNF waveguiding a 5-W-power 1552-nm-wavelength light. At each position (I: 0.5 cm, II: 1.0 cm and III: 1.5 cm away from the left side of the MNF), indicated by line segments beneath the MNF in the upper panel, a 570-μm-length segment of the MNF was imaged using a short-wave infrared camera (Goldeye, G-033 TECless) with dark background. b Corresponding grayscale statistical distribution of the surface scattering intensity. c Normal probability plot of the grayscale value at the position of 0.5 cm along the length of the MNF.

Supplementary Note 6. Self-cleaning effect in a high-power CW waveguiding MNF

The MNF waveguiding a high-power light enables a self-cleaning effect to clean off the adsorbate, as can be seen intuitively by the reduction of the scattering intensity of the probing light in Fig. S6. The high-power self-cleaning effect can clean the surface contamination (e.g., absorption centers on the fibre surface) and recover the degraded optical transmittance to a certain degree (e.g., ~90%). Note that usually the surface contamination cannot be removed completely by this way, which may gradually degrade the surface of a high-power waveguiding MNF under long-term operation. For long-term operation of the MNF with high optical power, a complete isolation of the MNF is necessary (e.g., sealed in a box filled with high-purity nitrogen gas in Fig. S4).


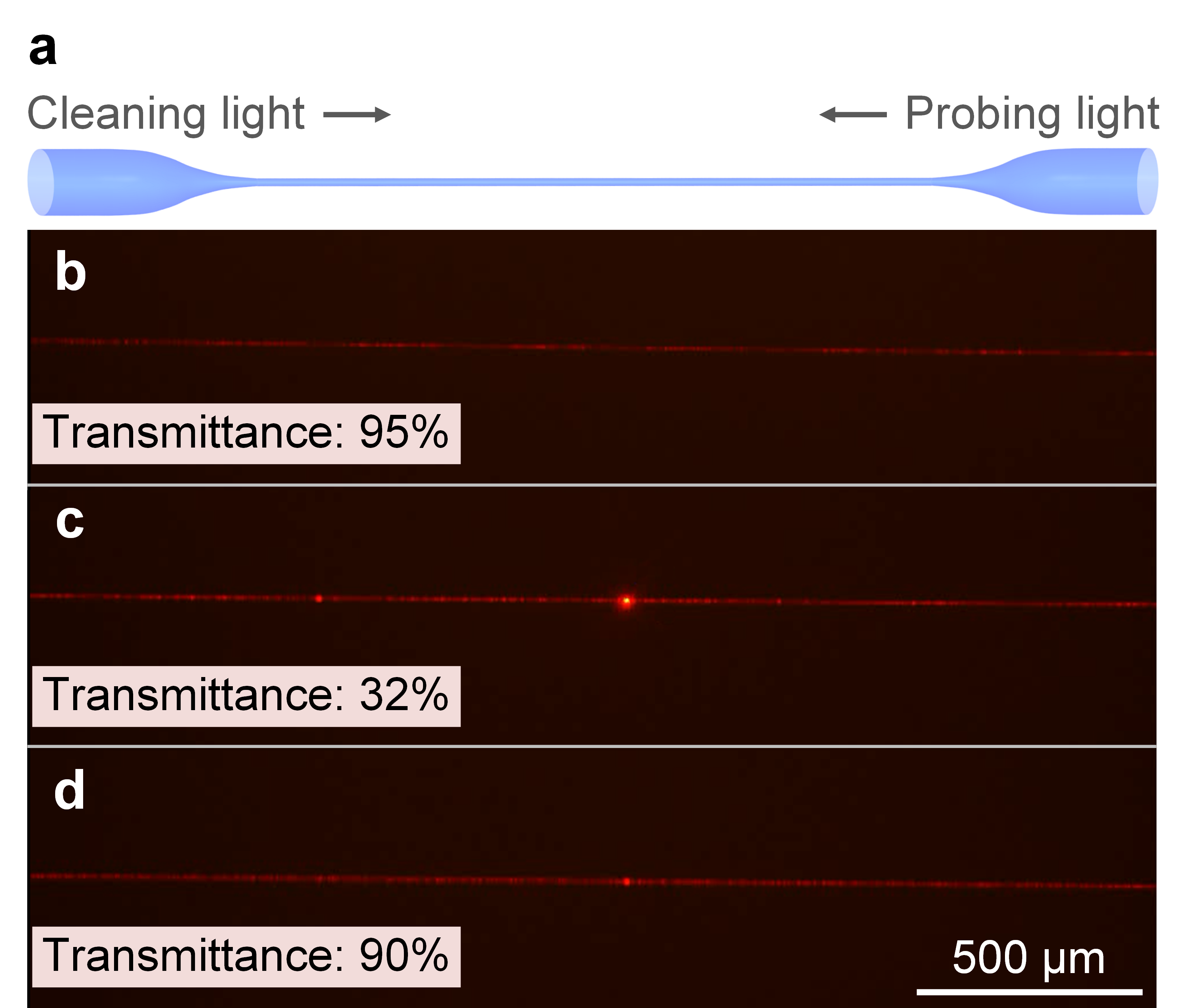


Fig. S6. Self-cleaning effect in a high-power waveguiding MNF. a Schematic diagram of optical characterization. A 1552-nm-wavelength high-power CW light was sent from the left side as a cleaning light, while a 5-mW-power 650-nm-wavelength CW probing light was sent from the right side and propagates oppositely. b-d Optical microscope images showing the surface scattering of the probing light waveguided along the length of a 1.1-μm-diameter MNF b before and c after adsorbing dusts without cleaning light, and d after a 13-W-power CW cleaning light was sent into the MNF from the left side for 7 s (video S1). For reference, the measured optical transmittance around 1550-nm wavelength was 95% in b, 32% in c and 90% in d, respectively, indicating an effective cleaning of the surface contamination to a certain degree.

Supplementary Note 7. Temperature measurement of a high-power CW waveguiding MNF

To precisely measure the temperature rising of a high-power waveguiding MNF, we assemble the MNF into a knot resonator^1^, and obtain the temperature by the power-dependent spectral shift of the resonant peak.

As the MNF temperature rises with increasing waveguiding power (via intrinsic and defect absorption), both the refractive index of the MNF (thermo-optic effect) and cavity length of the knot resonator (thermal expansion) increase, leading to a red shift of the resonant wavelength *λ*_res_ of the silica MNF knot resonator (SMKR) that can be obtained as^2^

(S1)

where Δ*λ*_res_ is the shift of *λ*_res_, Δ*T* is temperature change. For silica, thermo-optic coefficient *α* ≈ 8.1×10^-6^ ℃^-1^, and thermal expansion coefficient *β* ≈ 5.5×10^-7^ ℃^-1^ (refs. 3-5). We theoretically obtain Δ*λ*_res_/Δ*T* of 12.2 pm ℃^-1^ around 1550-nm wavelength.

Experimentally, we assembled a 910-nm-diameter MNF into a 360-µm-diameter SMKR, and placed it on the top of an electrically heated hotplate with temperature precisely controlled (Fig. S7a). Then we increased the temperature of the hotplate (also the MNF) from 22 ℃ to 300 ℃ step by step, and measured the spectral shift of the resonant peak using a tunable CW fibre laser (Santec, TSL-710) and a power meter (Santec, MPM-210). Based on the measured temperature-dependent resonant spectra that clearly showed the spectral shift of the resonant peak (Fig. S7b), we obtained an almost linear dependence of spectral shift of the resonant wavelength *λ*_res_ on the temperature *T* (Fig. S7c), and retrieved an experimental Δ*λ*_res_/Δ*T* of ~11.8 pm ℃^-1^, which agreed well with the theoretical value (i.e., 12.2 pm ℃^-1^). Based on the value of Δ*λ*_res_/Δ*T*, we can thus obtain power-dependent temperature in the MNF shown in Fig. 2c.


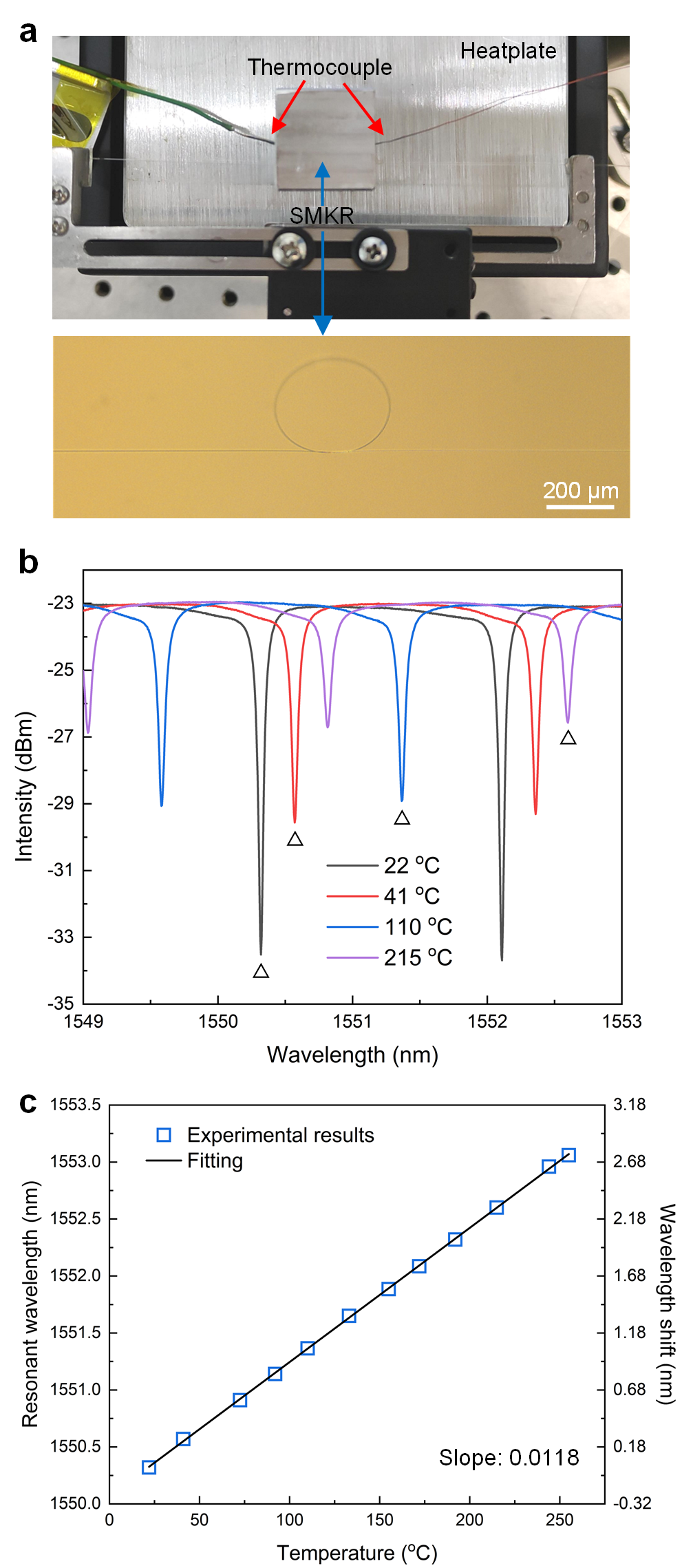


Fig. S7. Experimental measurement of temperature-dependent spectral shift of the resonant wavelength of a 360-µm-diameter SMKR assembled with a 910-nm-diameter MNF. a Experimental setup. b Typical results of the measured temperature-dependent resonant spectra, clearly showing the spectral shift of the resonant peak. c Temperature-dependent spectral shift of the resonant wavelength, retrieving an experimental Δ*λ*_res_/Δ*T* of ~11.8 pm ºC^-1^.

Supplementary Note 8. Dependence of optical transmittance on the temperature of the MNF


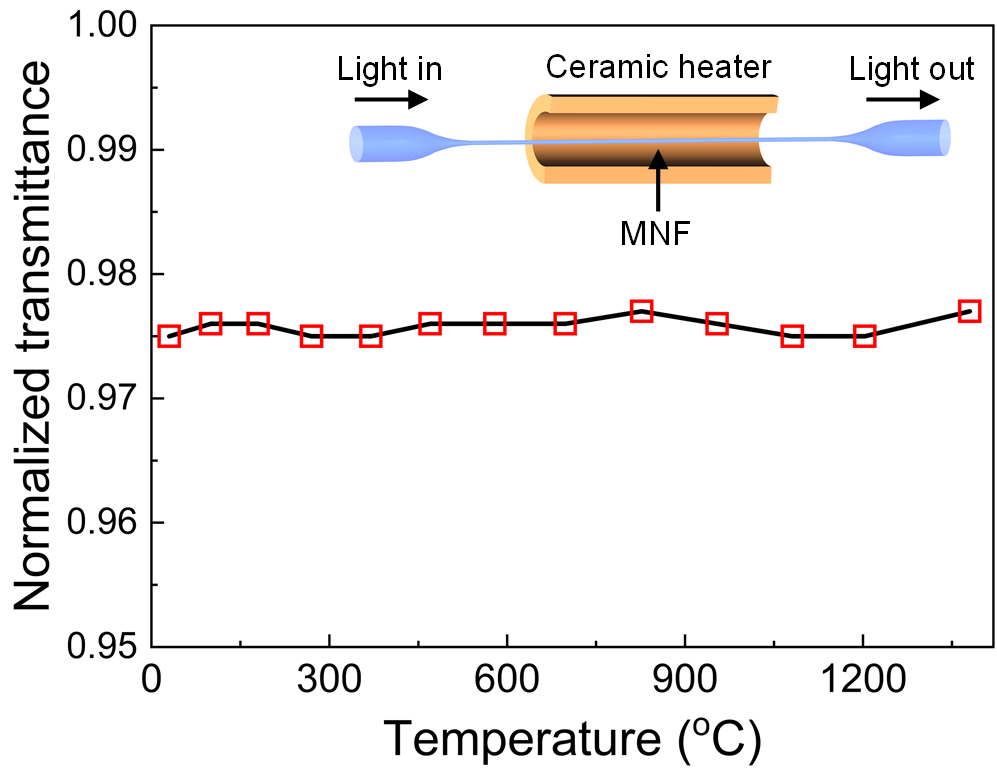


Fig. S8. Dependence of optical transmittance of the MNF on the temperature of the MNF. Inset, schematic diagram of the experimental test. In the test, a 1.2-μm-diameter 2-cm-length MNF was placed inside a ceramic heater, and the optical transmittance of the MNF was measured with a waveguided 1-mW-power CW 1550-nm-wavelength light. With temperature increasing from 28 ºC to ~1400 ºC, the optical transmittance kept almost a constant of ~97.5%.

Supplementary Note 9. Simulation of the temperature of a high-power CW waveguiding MNF

The MNF used in our simulation is 1.2 μm in diameter and 2 cm in length, surrounded by air at room temperature (22 ℃). The material parameters used here are listed below:

| Material | Thermal Conductivity (W m^-1^ K^-1^) | Heat Capacity (J kg^-1^ K^-1^) | Density (g cm^-3^) | Ref. |
| --- | --- | --- | --- | --- |
| Air | -2.3×10^-3^+1.2×10^-4^T-7.9×10^-8^T^2^  +4.1×10^-11^T^3^-7.4×10^-15^T^4^ | 1×10^3^-3.7×10^-1^T+9.5×10^-4^T^2^-  6.0×10^-7^T^3^+1.3×10^-10^T^4^ | 1.2×10^-3^ | 5 |
| Silica | 1.38 | 703 | 2.203 | 6 |


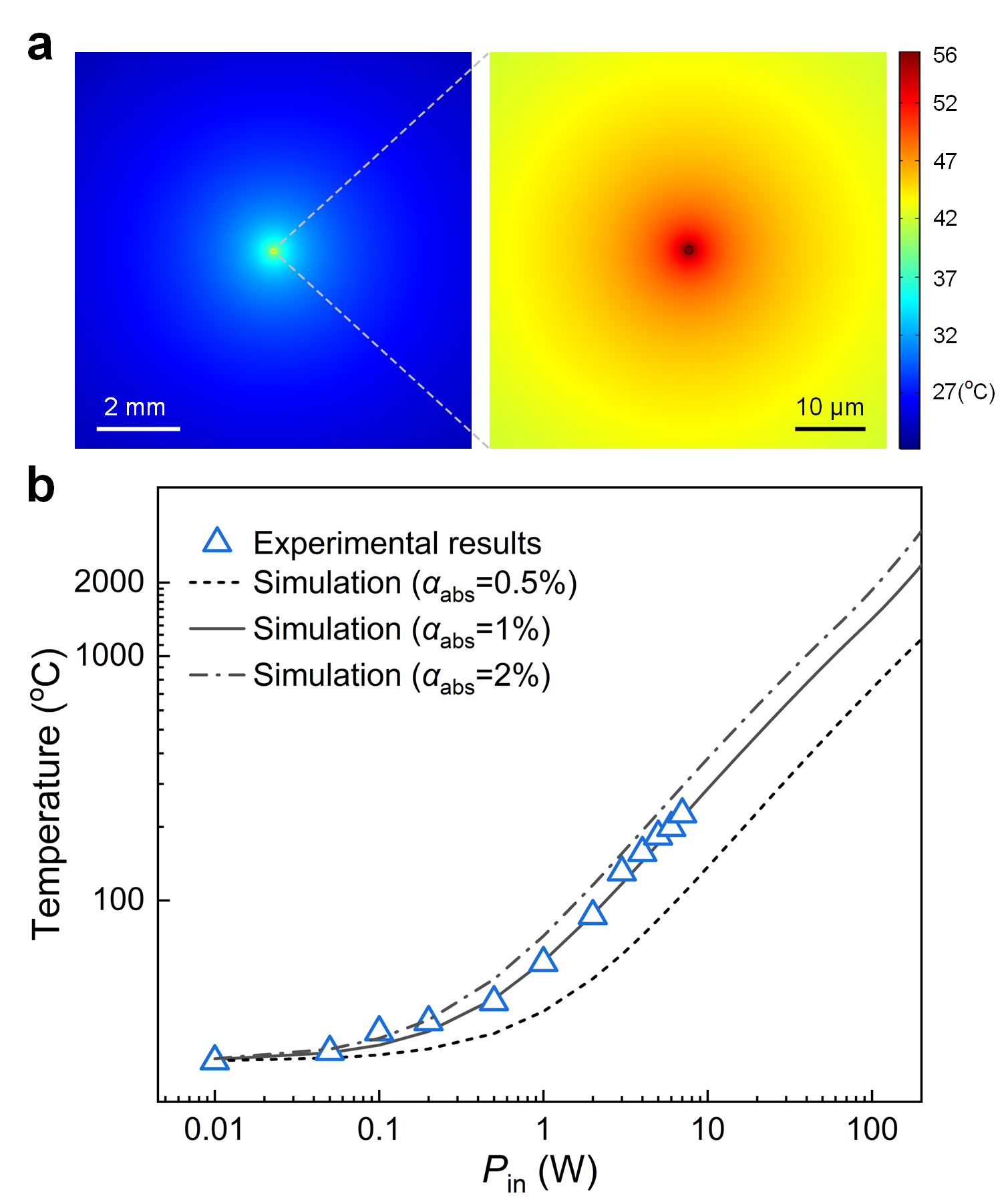


Fig. S9. Simulation of the temperature of a 1.2-μm-diameter 2-cm-length MNF. a Cross-sectional temperature distribution of the MNF waveguiding a 1-W-power 1550-nm-wavelength CW light (left). Detail is shown from the dash lines (right). b Waveguiding-power-dependent temperature of the MNF in a steady state with different absorption coefficients.

The thermal power for heating the MNF *P*_heat_ that is generated by absorbing waveguiding power *P*_in_ is *P*_heat_ = *P*_in_ × *α*_abs_, where *α*_abs_ is the overall absorption coefficient of the MNF. Based on the thermal dissipation simulation (Fig. S9a) of the MNF and experimentally measured power-dependent temperature results (Fig. 2c), we obtain the value of *α*_abs_ of ~1%.

Considering higher transmittance (e.g., > 99%) can be obtained in a MNF (i.e., lower absorption coefficient *α*_abs_)^7^, higher optical damage threshold of the MNF is expected to be achieved. For reference, in Fig. S9b, waveguiding-power-dependent temperature with two other values of *α*_abs_ (0.5% and 2%, respectively) are also provided, indicating that optical damage threshold as high as 180 W can be achieved if *α*_abs_ can be reduced to 0.5%.

Supplementary Note 10. Point defects in silica MNFs

Despite the ignorable intrinsic absorption of the MNFs around 1550-nm wavelength, the defect absorption may play a key role on the optical damage of a MNF, especially when operating at high power. During the fibre drawing process at high temperature, the six-membered rings of the amorphous silica are likely to deform to three-membered rings (i.e., strained Si-O-Si bonds), as the precursor sites for Si and O dangling bonds, i.e., E’-center and non-bridging oxygen hole center (NBOHC)^8-11^. Heating under oxygen-deficient conditions also causes diamagnetic oxygen deficient centers (ODCs, e.g., ODC(Ⅰ) and ODC(Ⅱ)) on the MNF surface. Other types of derived defects, e.g., peroxy radical (POR) and interstitial O_2_, are thermally activated with temperature > 200 ℃. High-intensity light irradiation on the MNFs efficiently promotes the formation of surface dangling bonds and ODCs, together with emissions of the photoluminescence (PL) bands.

Experimentally we used an excitation light from a 405-nm-wavelength CW laser (Oxlasers, OX-DK4055) to examine the defects on the MNF surface, as schematically illustrated in Fig. S10a. After passing through a beam expander (i.e., a cylindrical lens), the excitation light focused onto the surface of the MNF with a full length of ~2 mm and width of ~20 μm. The PL emission was coupled into the MNF, propagating along both sides of the MNF (Fig. S10b). After filtering out the input light, we obtained the PL emission signals by a spectrometer (Ocean Optics, USB2000+). The PL spectra in Fig. S10c show two PL bands centered around 1.9 eV and 2.7 eV, corresponding to the defect luminescence of NBOHC and ODC(Ⅱ). Due to electron-phonon coupling, the PL spectra are broadband^8^. When waveguiding high-power CW light around 1550-nm wavelength, such point defects on the MNF surface will give rise to a detrimental photothermal effect by multiphoton absorption^12^.


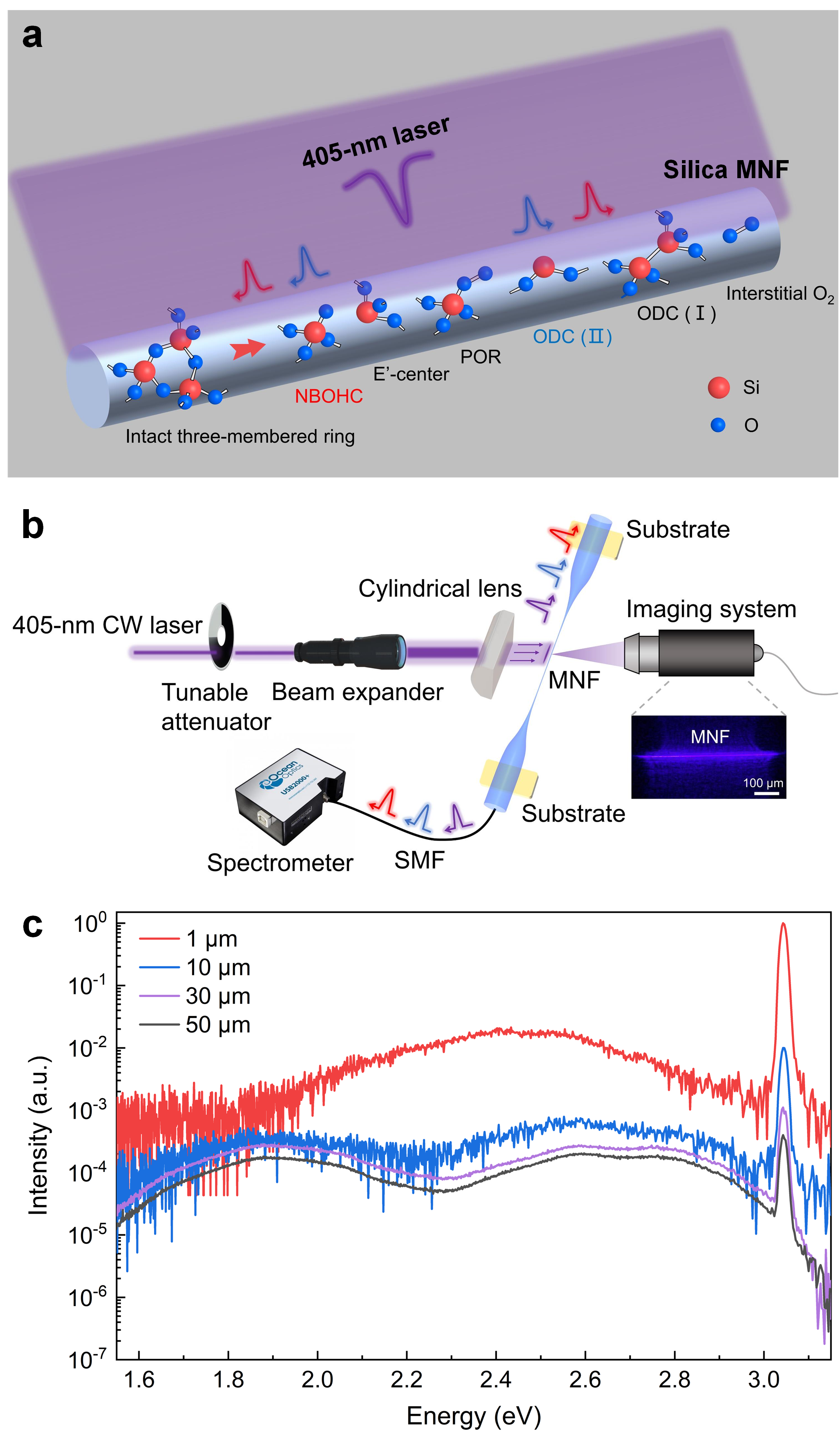


Fig. S10. Point defects in silica MNFs. a Schematic illustration of point defects in silica MNFs and PL characterization using a 405-nm-wavelength excitation light. NBOHC, non-bridging oxygen hole center; POR, peroxy radical; ODC, oxygen deficient center. b Schematic illustration of experimental setup for PL excitation and measurement. Inset, optical microscopy image collected by the imaging system. The MNF was suspended in the air and located at the focal plane of the cylindrical lens. c PL spectra of the MNFs with different diameters. The peak at 3.05 eV corresponds to the excitation light.

Supplementary Note 11. OH^-^ absorption of MNFs around 1550-nm wavelength

As the fibre diameter decreases, the enhanced surface field (i.e., evanescent field) and surface-to-volume ratio increase the absorption of surrounding water molecules in the vicinity of the MNF surface. Previous results in Fig. S1b indicate that the optical absorption of OH^-^ is centered around 1400-nm wavelength, close to the 1550-nm transmission window. To get more details of the OH^-^ absorption in the near-infrared spectrum region, we immersed the MNF in deionized water and measured the optical absorption of the OH^-^ when the MNF was waveguiding light from a broadband light source (Thorlabs, SLS201L). To eliminate additional transmission loss induced from mode leakage of the MNFs in the water, the MNF diameters used here were 2 μm and 5 μm, respectively. As shown in Fig. S11, the OH^-^ absorption in the MNFs covers a relatively wide spectral range from ~1400-nm to ~1700-nm wavelength, and stronger absorption occurs in a thinner MNF. Therefore, in a MNF waveguiding high-power CW light around 1550 nm, the OH^-^ absorption could also heat the MNF and thus be a factor that determined the optical damage threshold of the MNF.


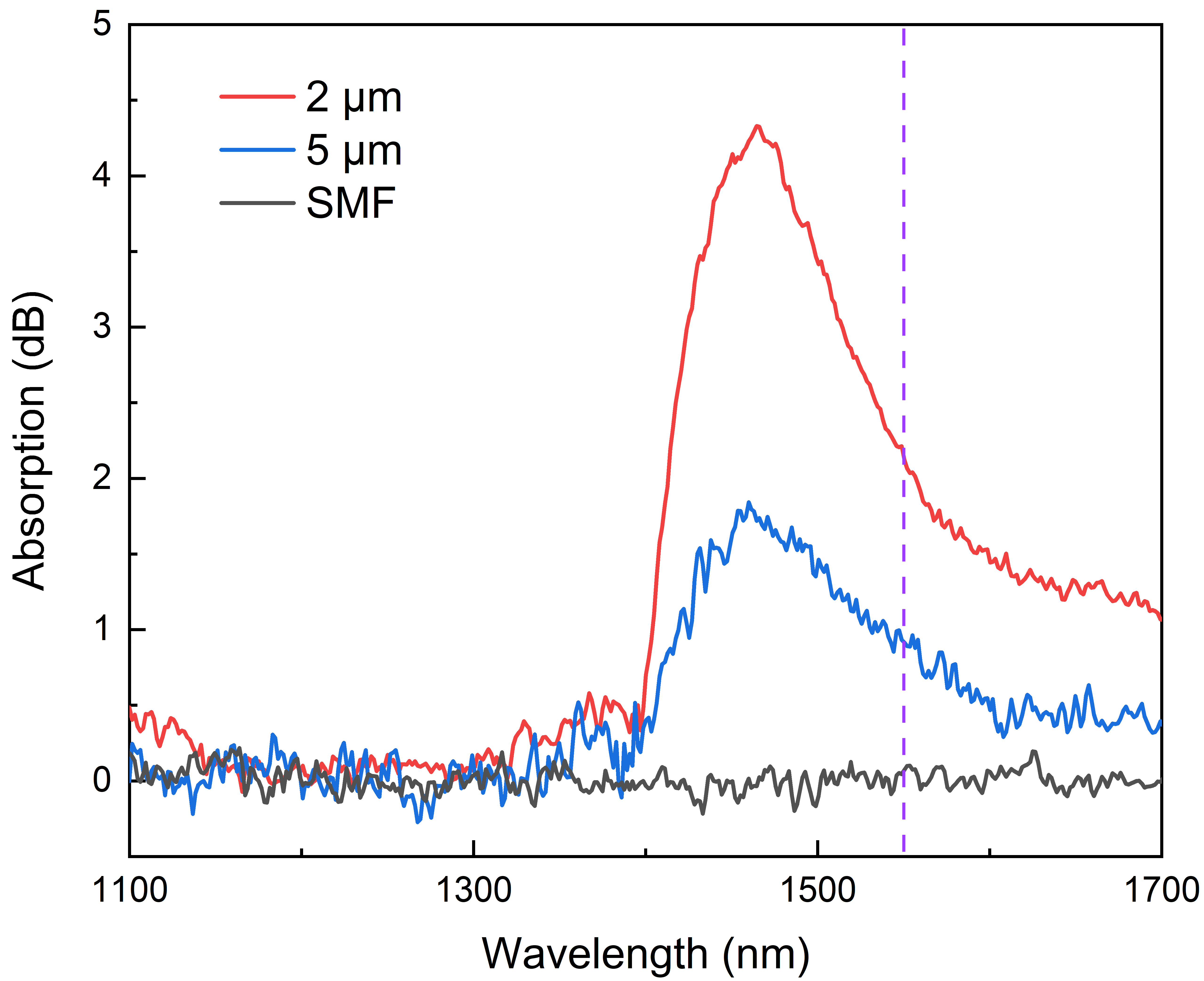


Fig. S11. OH^-^ absorption spectra of 2-cm-length MNFs with diameters of 2 μm and 5 μm, respectively. For reference, absorption spectrum of a standard single-mode fibre (SMF) is also provided.

Supplementary Note 12. Calculation of the optical force on the oil droplet along the MNF

By using finite-difference time-domain (FDTD) simulation, the electric field distribution around the 1-μm-diameter MNF was obtained and the optical force on the oil droplet (11 μm × 10 μm ellipsoid) was calculated.

The optical force *F* on the oil droplet can be calculated from the Maxwell stress tensor *T*, given by^13^

$\mathbf{F}=\int_{S} \left\langle\mathbf{T}\right\rangle\cdot\mathbf{n}_{S}dS$ (S2)

where *n*_S_ is a normal vector pointing in the outward direction from the surface *S*, and the elements of the Maxwell stress tensor *T*_i,j_ can be expressed as

$\mathbf{T}_{i,j}=\varepsilon_{0}\varepsilon_{r}\mathbf{E}_{i}\mathbf{E}_{j}+\mu_{0}\mu_{r}\mathbf{H}_{i}\mathbf{H}_{j}-\frac{1}{2}\delta_{i,j}(\varepsilon_{0}\varepsilon_{r}\left|\mathbf{E}\right|^{2}+\mu_{0}\mu_{r}\left|\mathbf{H}\right|^{2})$ (S3)

where subscripts *i* and *j* are the indices running from *x*, *y* to *z* in a Cartesian space, *ε*_0_ and *µ*_0_ are the permittivity and permeability of vacuum, *ε*_r_ and *µ*_r_ are the relative permittivity and relative permeability of the medium, *E* and *H* are the electric field and magnetic field vectors, *E*_i_ and *E*_j_ are the *i*-th and *j*-th components of *E* vector while *H*_i_ and *H*_j_ are the *i*-th and *j*-th components of *H* vector, *δ*_i,j_ is Kronecker's delta.

In our simulation, the wavelength of the propagating light was set to be 1552 nm. The refractive indices of the MNF, air and the oil droplet were 1.444, 1 and 1.404, respectively. Based on equations (S2) and (S3), the axial optical force *F*_x_ exerted on the particle can be obtained. Figure S12 shows that the calculated axial optical force *F*_x_ on the oil droplet grows linearly with the waveguided power of the MNF, with a value of 0.85 nN W^-1^.


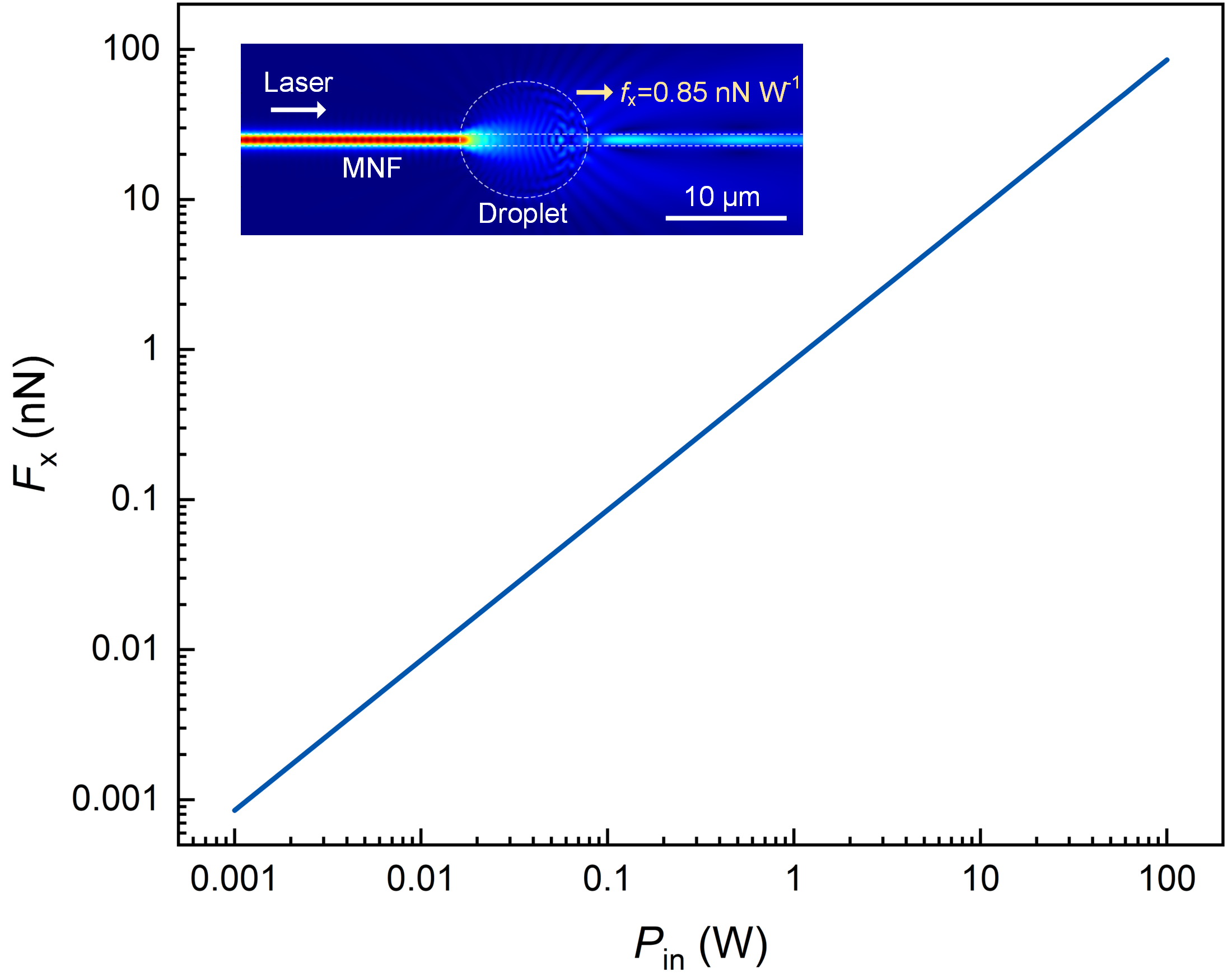


Fig. S12. Power-dependent axial optical force of the oil droplet along a 1-μm-diameter MNF. The axial optical force (*f*_x_=0.85 nN W^-1^) of the oil droplet grows linearly with the waveguided power.

Supplementary Note 13. Optical driving of a silica microsphere in air


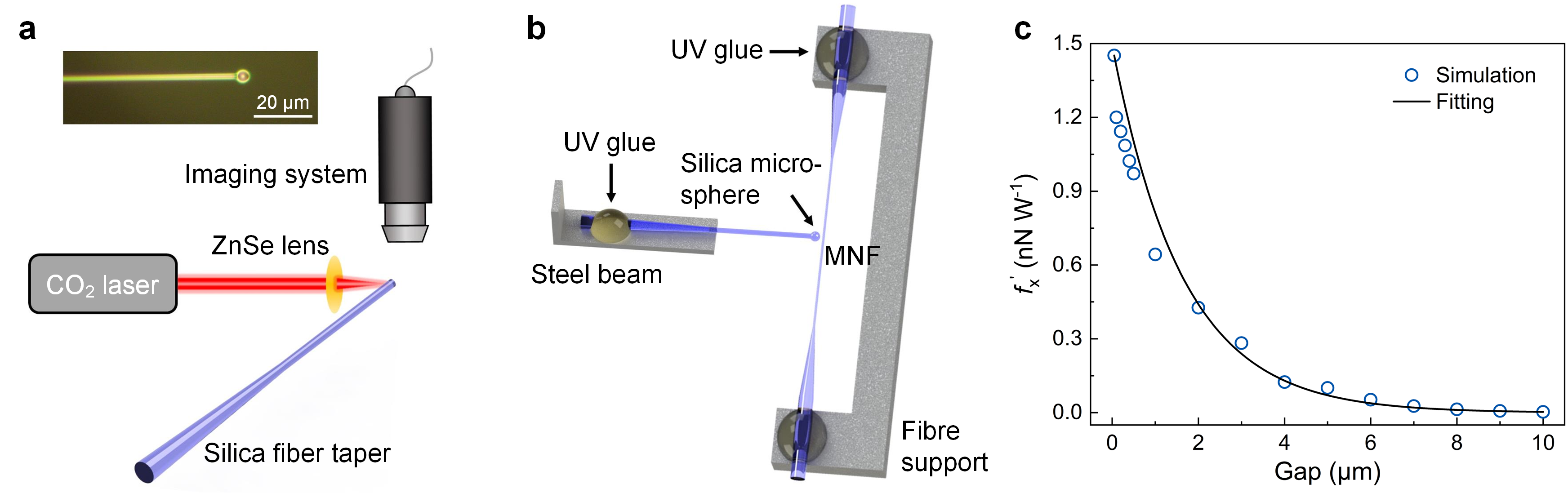


Fig. S13. Optical driving of a microsphere suspended in air. a Fabrication of the silica microsphere by melting the end of a silica fibre taper using a focused CO_2_ laser beam. Inset, optical microscope image of an as-fabricated microsphere attached to the fibre taper. b Schematic illustration of precisely controlling the gap distance between a microsphere and a MNF. The MNF mounted on a fibre support and the microsphere mounted on a steel beam were mounted on 3-D linear translation stages, respectively. The microsphere was finely tuned to move approaching the MNF and the gap distance between the microsphere and MNF was monitored by optical transmission of the MNF around 1550-nm wavelength. c Calculated power-dependent axial force (*f*_x_') exerted on the microsphere versus the gap distance. The diameter of the MNF used here is 410 nm.

Supplementary Note 14. Displacement of a silica microsphere driven by a waveguiding MNF


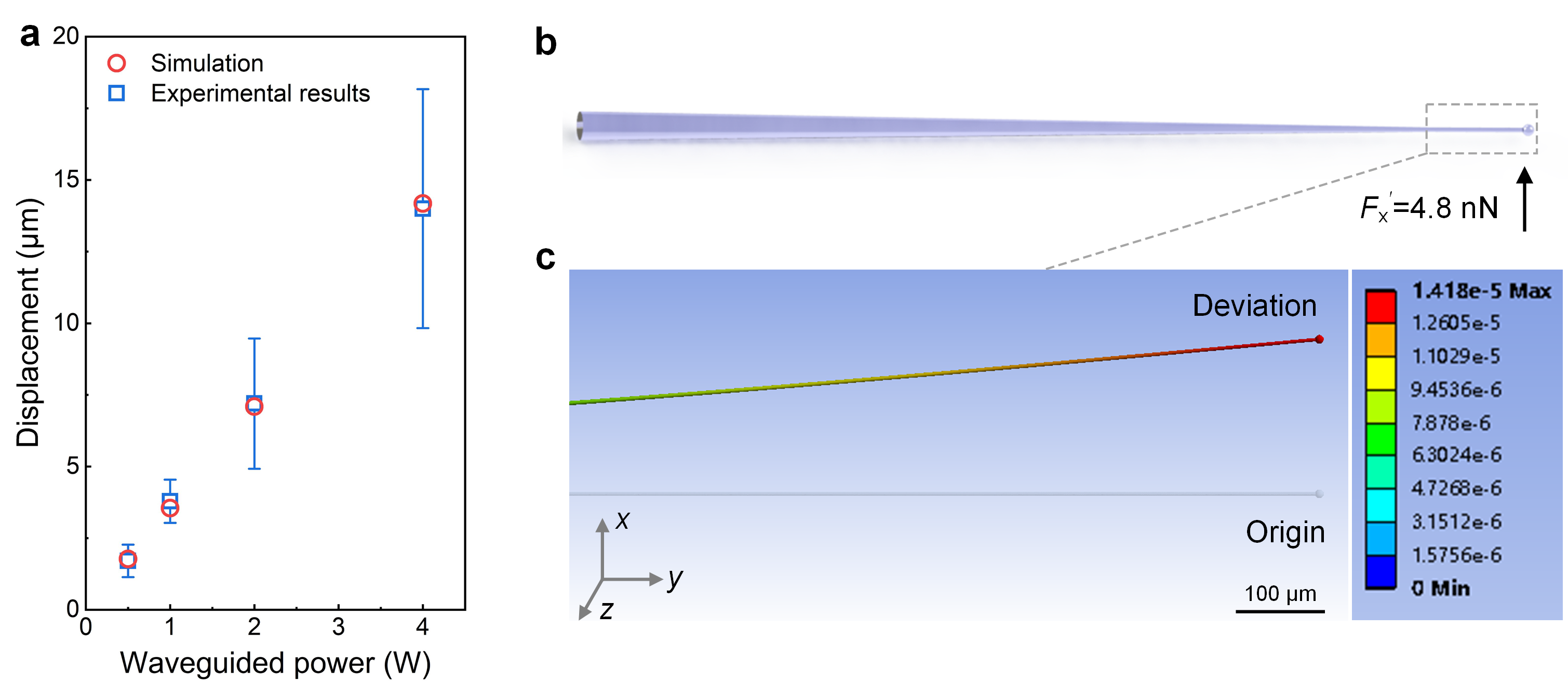


Fig. S14. Displacement of the microsphere under an axial optical force. a Experimental results of the displacement of the silica microsphere versus waveguided power of a 410-nm-diameter MNF, agreeing well with the simulation results. The simulation is performed with Ansys Workbench. b Schematic diagram of the microsphere and its supporting fibre taper. c Calculated displacement of 14.2 μm in the *x* direction of the microsphere driven by an optical force *F*_x_′=4.8 nN with a 4-W waveguiding power.

Supplementary Note 15. Intermodal phase matching and overlap integral for THG in MNFs

The effective refractive indices of waveguiding modes are calculated with exact solutions of Maxwell’s equations^14^ (Fig. S15a), and the values of overlap integral *J_3_* between the fundamental and the phase-matched modes are calculated by^15^

(S4)

where *F*_1_(x,y) and *F*_3_(x,y) denote the transverse electric mode field distributions of the fundamental and the TH modes, respectively. We calculate the electric fields of the phase-matching involved modes in the MNFs at the marked diameters and deduce the overlap integrals *J*_3_ from the equation (S4), as indicated in Fig. S15b. The results suggest that the most efficient overlap between the phase-matching modes is achieved for the combination of HE_11_(*ω*) and HE_12_(3*ω*) at the diameter of 768 nm, which is also intuitively discernible from the electric field distributions.


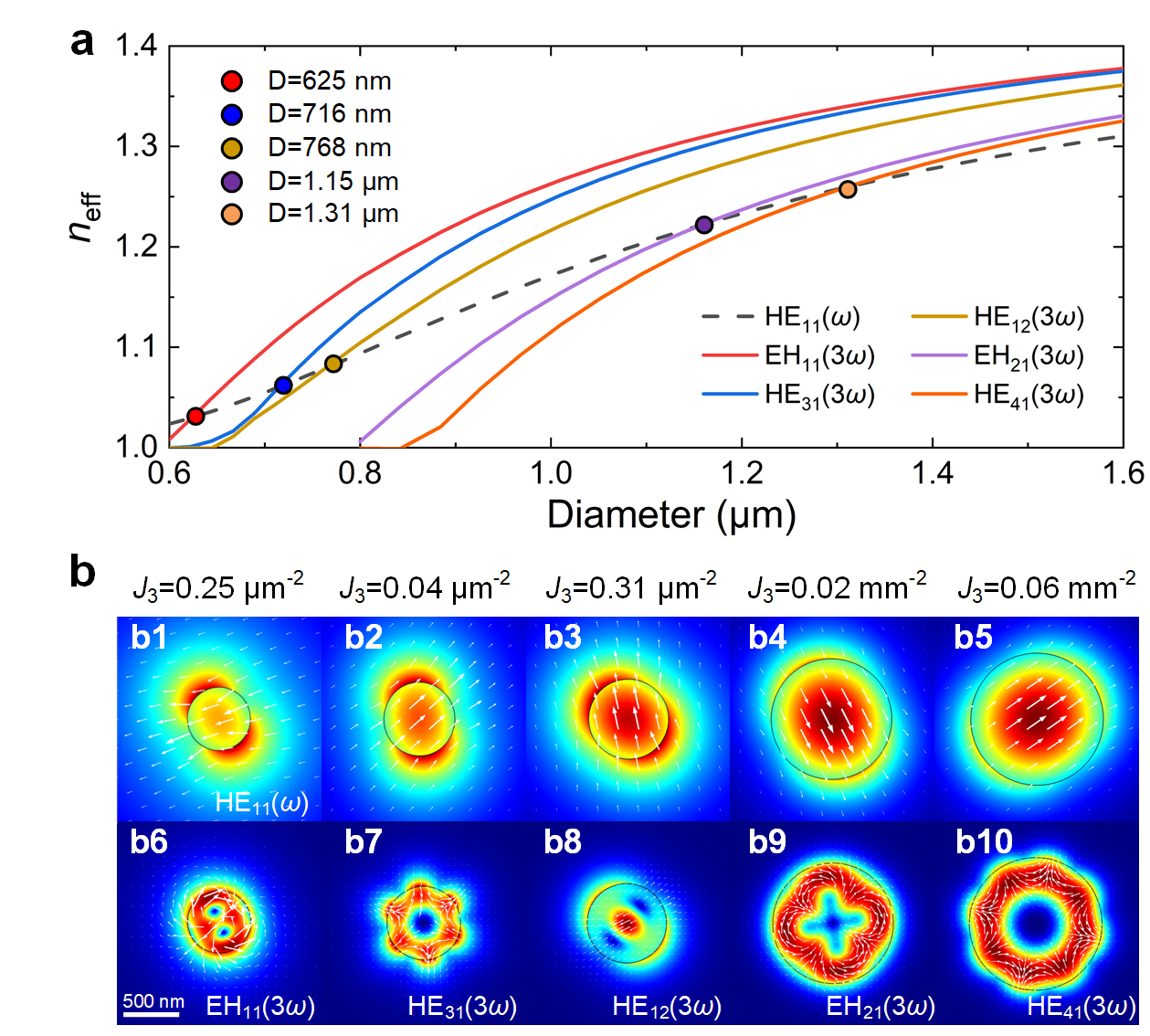


Fig. S15. Calculated intermodal phase matching condition and overlap integral for THG in MNFs with 1550-nm-wavelength fundamental light. a Dependence of effective refractive indices (*n*_eff_) of waveguiding modes on the diameter of the MNF. The right MNF diameters for intermodal phase matching are indicated by circles. b Electric fields and calculated values of overlap integral *J*_3_ of the phase-matched modes presented in a.

Supplementary Note 16. Intermodal phase matching for SHG in MNFs

For the SHG process, the second-order nonlinearity mainly arises from the surface nonlinearity presented at the glass/air interface of the MNF. Figure S16a shows the dependence of effective refractive indices of several modes on the SHG process in the MNFs. It is obvious that the MNF diameters for perfect intermodal phase matching are 608 nm (HE_11_(*ω*) and TE_01_(2*ω*)), 692 nm (HE_11_(*ω*) and TM_01_(2*ω*)) and 774 nm (HE_11_(*ω*) and HE_21_(2*ω*)). The electric fields of the phase-matching modes in the MNFs are shown in Fig. S16b. Similarly, the overlap integral *ρ*_2_ between the fundamental and the SH modes can be calculated by^16^

$\rho_{2}\text{=}\frac{\omega_{2}}{2A_{1}^{2}}\frac{\int dr_{\perp}e_{2}^{*}\cdot P^{(2)}}{\mathrm{Re}\int dr_{\perp}\left[e_{2}^{*}\times h_{2}\right]_{z}}$ (S5)

where *ω*_2_ is the second-harmonic frequency, *A*_1_ is the field amplitude of the fundamental light, *P*^(2)^ is the second-order nonlinear polarization (including both dipole contributions from the surface and multipole contributions from the bulk), *r*_⊥_ is the vector normal to the surface, and the electric and magnetic fields of the SH modes are expressed as $E(r,\omega_{2})=A_{2}e_{2}(r_{\perp},\omega_{2})\exp(i(\beta_{2}z-\omega_{2}t))$ and $H(r,\omega_{2})=A_{2}h_{2}(r_{\perp},\omega_{2})\exp(i(\beta_{2}z-\omega_{2}t))$, *A*_2_ and *β*_2_ are the field amplitude and the propagation constant of the SH signal.


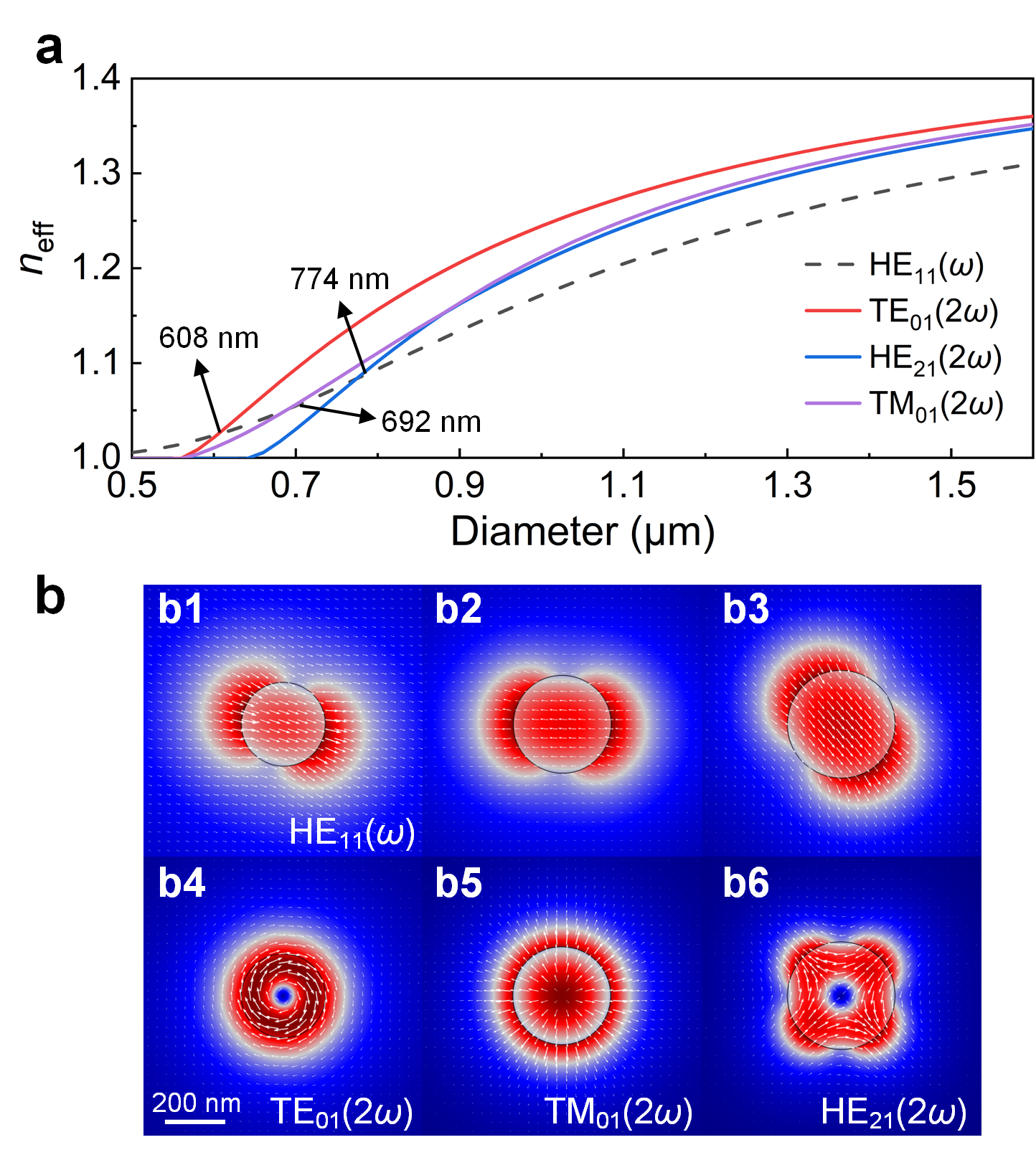


Fig. S16. Intermodal phase matching of the SHG in the MNFs with 1550-nm-wavelength fundamental light. a Dependence of effective refractive indices (*n*_eff_) of the fundamental and the SH modes on the MNF diameter at 1550-nm wavelength. The MNF diameters for intermodal phase matching in the SHG process are indicated. b Electric field simulations of the phase-matching modes at diameters of 608 nm (b1, b4), 692 nm (b2, b5) and 774 nm (b3, b6).

Supplementary Note 17. Fabrication of MNFs with high-precision diameter control

During the fabrication process, the real-time MNF diameter can be obtained by monitoring the cut-off of a certain high-order propagation mode (leading to a sudden drop in the total transmission) at a specific wavelength^17^. For a particular MNF diameter *D*_co_, the cut-off wavelength *λ*_co_ of the TE_01_ mode can be obtained as

$\lambda_{\text{co}}=\frac{\pi\sqrt{n_{1}^{2}-n_{2}^{2}}}{V}D_{co}$ (S6)

where *V*=2.405 is the normalized frequency, *n*_2_=1 is the refractive index of air, and *n*_1_ is the refractive index of the silica which follows Sellmeier-type dispersion formula (the unit of *λ* is μm)

$n_{1}^{2}(\lambda)=\frac{0.6961663\lambda^{2}}{\lambda^{2}-0.0684043^{2}}+\frac{0.4079426\lambda^{2}}{\lambda^{2}-0.1162414^{2}}+\frac{0.8974794\lambda^{2}}{\lambda^{2}-9.896161^{2}}+1$ (S7)

Thus, we can preset an arbitrary target diameter as the cut-off diameter of the TE_01_ mode, monitor the cut-off signal in the real-time transmission at the wavelength calculated from equation (S6), and stop the pulling process at the target diameter (e.g., monitoring the TE_01_ mode at 1055-nm wavelength for the cut-off diameter of 780 nm). In the experiment, we used a white light source (Energetiq, EQ-99 LDLS) with a spectrum ranging from 190 nm to 2100 nm to monitor the cut-off of the high-order modes. As shown in Fig. S17a, there are 5 drops in the 1055-nm-wavelength transmission spectrum, corresponding to the cut-offs of EH_21_, EH_11_, HE_12_, HE_21_ and TE_01_ modes, respectively. When the MNF diameter decreases to ~780 nm, the cut-off of the TE_01_ mode happens (i.e., the rightmost drop). Once the cut-off signal of the TE_01_ mode is observed, the MNF pulling process is stopped instantly. Note that the as-fabricated MNF maintains a high optical transmittance of 95% around 1550-nm wavelength. The diameter measurement of a typical as-drawn MNF is given in Fig. S17b, showing an excellent diameter uniformity with diameter fluctuation less than ±2 nm within the measured fibre length of 4 cm.


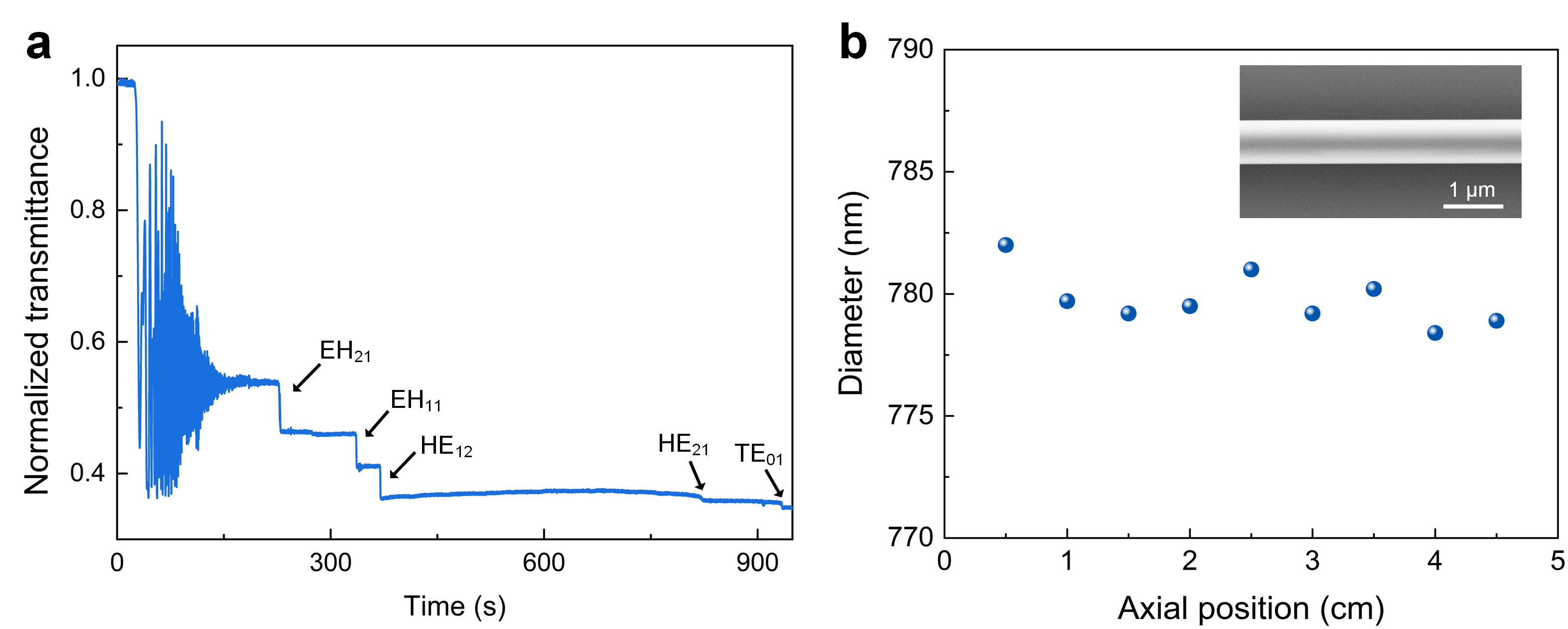


Fig. S17. High-precision diameter control of a MNF based on real-time feedback from high-order mode cut-off. a Real-time transmission measurement at 1055-nm wavelength during the pulling process. The cut-off diameter of the TE_01_ mode at 1055-nm wavelength is 780 nm. b Measured waist diameters of an as-fabricated 7-cm-length MNF along its axis.

Supplementary Note 18. Experimental measurement of the CW nonlinear harmonic generation

The experiment setup is shown in Fig. S18. The seed light from a tunable CW fibre laser (Santec, TSL-710) was amplified by an EDFA (Connet, MFAS-Er-C-B-15) with a maximum gain of 41.5 dB. The MNF was sealed inside a clean box to prevent possible surface contamination. The input end of the MNF (i.e., a piece of untapered standard fibre) was fusion spliced to the output fibre of the EDFA, and the output end of the MNF (i.e., another piece of untapered standard fibre) was fusion spliced to a short-pass fibre-based filter formed by wrapping a 4.5-m-length fibre around a 2-cm-diameter copper rod. The 1550-nm-wavelength light was filtered out via bending loss (> 50 dB around 1550-nm wavelength), while harmonic signals maintained relatively high transmission (bending loss < 3 dB). The harmonic signals were measured by a spectrometer (Ocean Optics, USB2000+) for spectral analysis and by a power meter (Thorlabs, PM100D and S120C) for determining the absolute optical power.


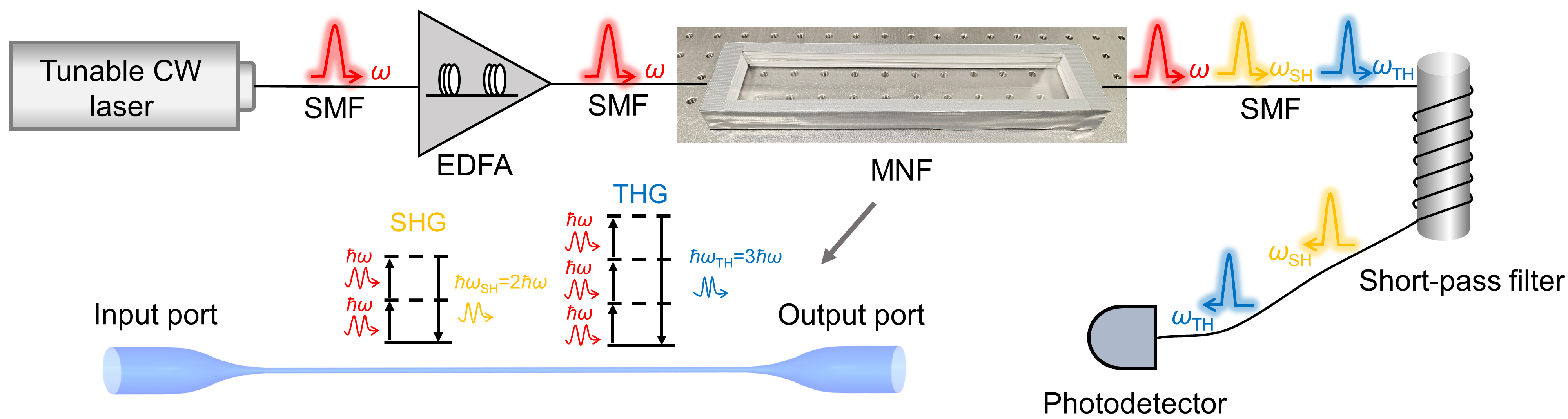


Fig. S18. Schematic diagram of experimental setup for CW nonlinear harmonic generation and measurement in a MNF. Inset, schematic illustration of the SHG and THG processes in a MNF. *ω*_SH_, second-harmonic frequency; *ω*_TH_, third-harmonic frequency.

Supplementary Note 19. Spectra of the high-power CW light


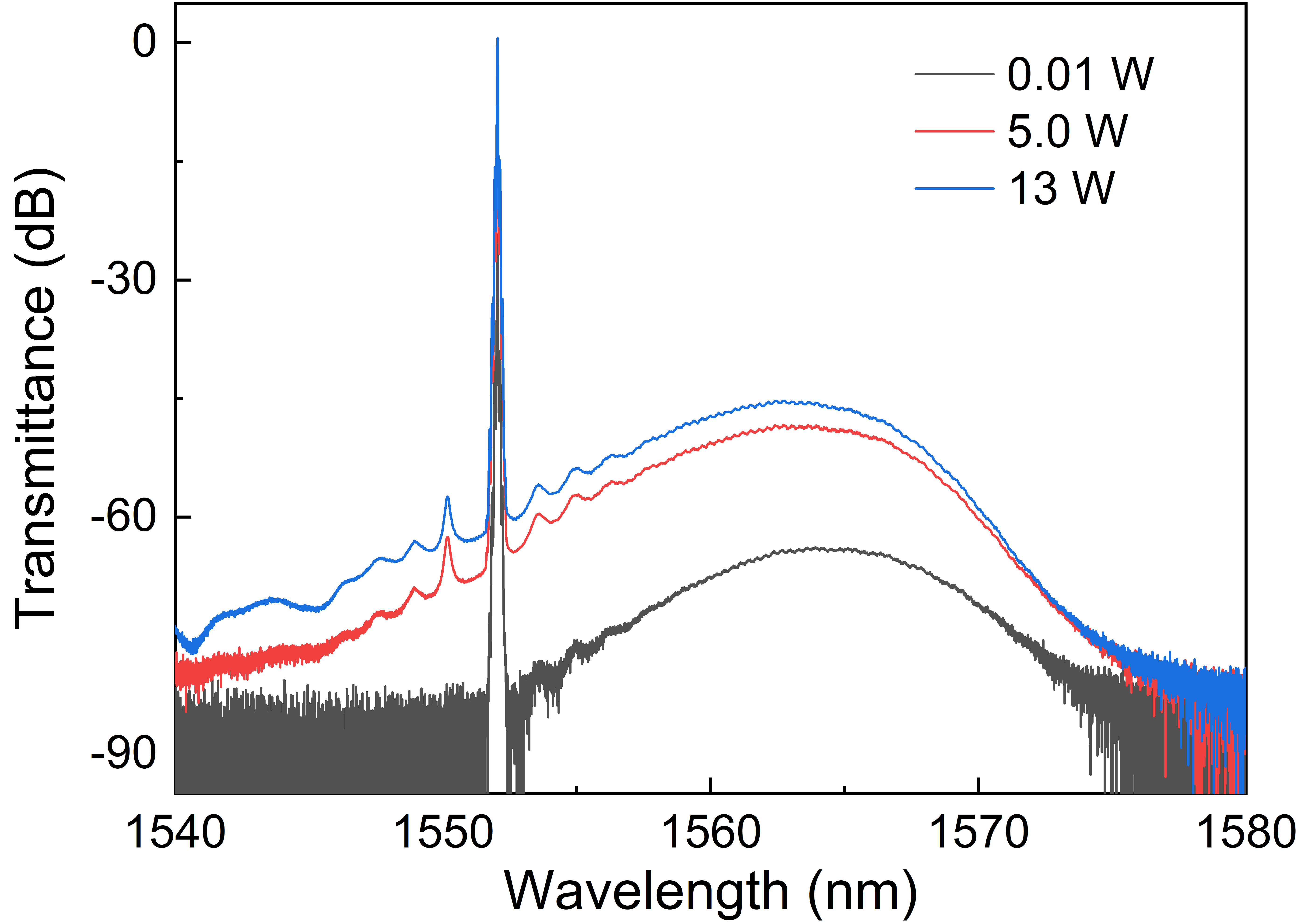


Fig. S19. Spectra of the high-power CW light output from the EDFA at three different powers of 0.01, 5.0 and 13 W, respectively. Although the EDFA (Connet, MFAS-Er-C-B-15) used in this work has a broad high-gain bandwidth, the spectra of the high-power CW light (e.g., at 1552-nm wavelength) after amplified by the EDFA maintains a good fidelity of the seed laser (Santec, TSL-710), with the dominant peak over 40 dB higher than the background.

Supplementary Note 20. Nonlinear effects in a standard silica fibre

In the experiment, we have also examined the possible existence of nonlinear optical effects in a standard silica fibre before pulling into a MNF. As shown in Fig. S20, with 8-W waveguiding power, the output spectrum of the MNF shows evident TH and SH signals, while that of an untapered 20-cm-length standard fibre (Corning, SMF-28e) shows no signal within the same spectral range, confirming that the nonlinear effects in the standard optical fibre in our work is negligible.


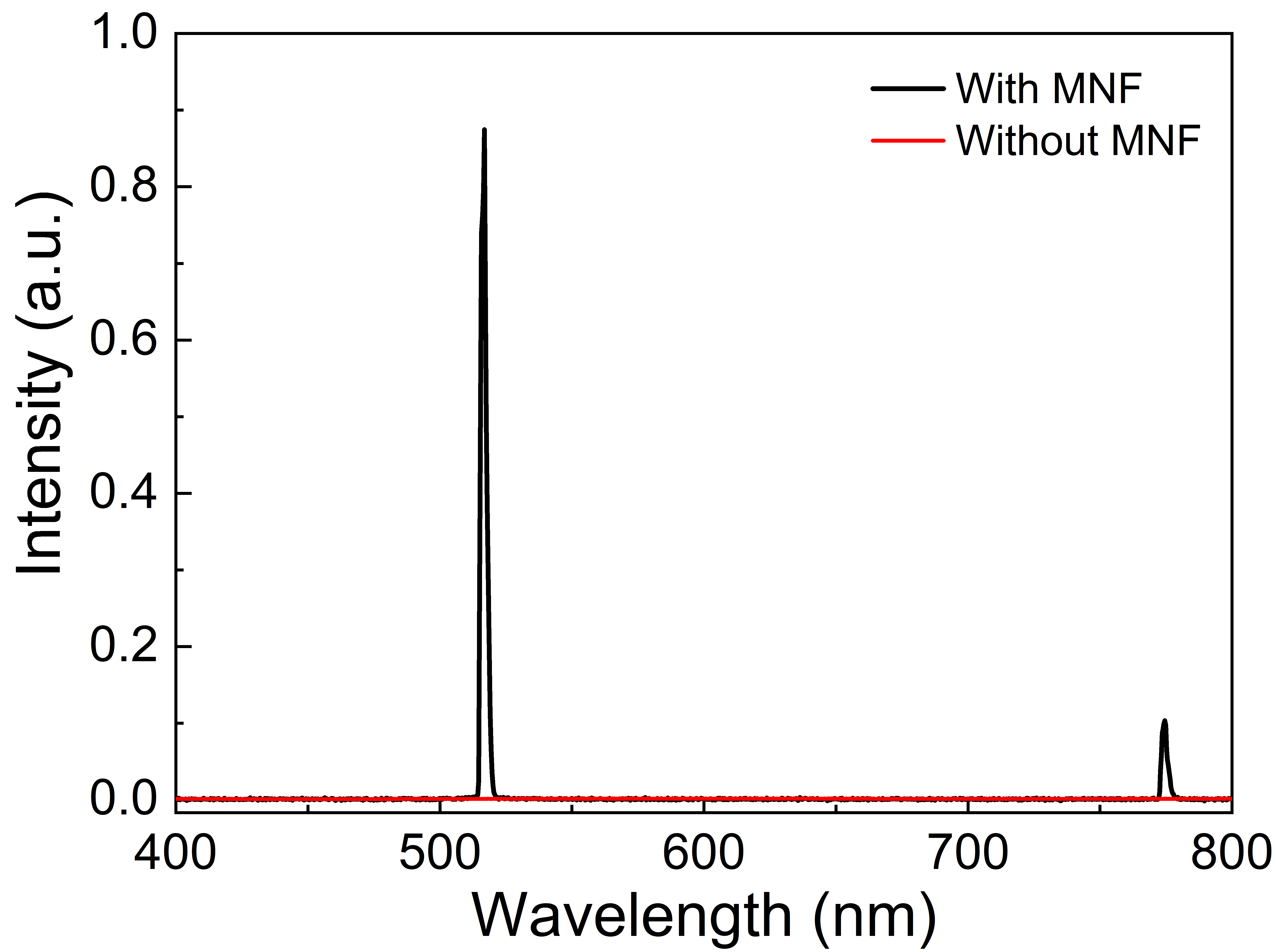


Fig. S20. Output spectra of a 20-cm-length untapered standard fibre and a 790-nm diameter, 9-cm-length MNF with a CW waveguiding power of 8 W.

Supplementary Table S1. Typical conversion efficiency of harmonic generation in MNFs and several other types of fibres

| Material | Geometry | Nonlinear process | Diameter (μm) | Interaction length (mm) | *λ*_ω_ (nm) | Pulse width | *P*_peak_ (W) | *ξ* | Year | Ref. |
| --- | --- | --- | --- | --- | --- | --- | --- | --- | --- | --- |
| SiO_2_ | MNF | *χ*^(3)^, THG | 2.6 | 90 | 1250 | 30 fs | 1.0×10^4^ | 5.0×10^-4^ | 2003 | 18 |
| SiO_2_ | MNF | *χ*^(3)^, THG | 0.5 | 0.1 | 1064 | 30 ns | 250 | 2.0×10^-6^ | 2007 | 19 |
| SiO_2_ | MNF | *χ*^(2)^, SHG | 0.408 | 4 | 940 | CW | 0.12 | ~10^-8^ | 2010 | 20 |
| SiO_2_  SiO_2_ | MNF  MNF | *χ*^(2)^, SHG  *χ*^(3)^, THG | 0.6  0.6 | 10  10 | 1555  1555 | 400 ps  400 ps | 1500  1500 | 2.0×10^-9^  2.5×10^-7^ | 2012  2012 | 21  21 |
| SiO_2_ | MNF | *χ*^(3)^, THG | 2.1 | 45 | 1550 | 4 ns | 1250 | 3.0×10^-4^ | 2012 | 22 |
| SiO_2_ | MNF | *χ*^(2)^, SHG | 0.7 | 0.2 | 1550 | 5 ns | 90 | 2.5×10^-9^ | 2013 | 23 |
| SiO_2_ | MNF | *χ*^(3)^, THG | 0.5 | 6 | 1091 | 2.3 ns | 2000 | 4.0×10^-7^ | 2019 | 24 |
| SiO_2_  SiO_2_ | MNF  MNF | *χ*^(2)^, SHG  *χ*^(3)^, THG | 0.779  0.779 | 70  70 | 1558.2  1572.5 | CW CW | 11.3  11.3 | 8.2×10^-8^  4.9×10^-6^ | 2023  2023 | This work  This work |
| SiO_2_ | PCF | *χ*^(2)^, SHG | / | 150 | 800 | 120 fs | 3.9×10^4^ | 1.6×10^-6^ | 2016 | 25 |
| SiO_2_ | PCF | *χ*^(3)^, THG | / | 100 | 1511 | 18 ps | 5556 | 2.6×10^-6^ | 2016 | 26 |
| Tellurite | SF | *χ*^(3)^, THG | 125 | 100 | 1500-1680 | 1 ps | 5.0×10^6^ | 1.0×10^-3^ | 2011 | 27 |
| Chalcogenide-tellurite | SF | *χ*^(3)^, THG | 120 | 30 | 1700-2600 | 200 fs | 6900 | 1.8×10^-13^ | 2014 | 28 |
| Tellurite | SF | *χ*^(2)^, SHG | 125 | 2500 | 1545 | 4.1 ns | 195 | 1.2×10^-2^ | 2019 | 29 |
| The table shows the performance of the present fibre-based optical frequency-conversion devices. It contains two parts, a review of the silica-MNF-based optical frequency-conversion device over the years (upper) and other fibre-based devices assisted with different materials and geometry (bottom). *λ*_ω_, fundamental wavelength; *P*_peak_, peak power of the pulse laser; *ξ*, conversion efficiency; PCF, photonic crystal fibre; SF, standard fibre. | | | | | | | | | | |

Captions for video files

Supplementary Video S1: Self-cleaning effect in a high-power CW waveguiding MNF.

Supplementary Video S2: Optomechanical manipulation of an oil droplet along a MNF, with a CW waveguided power increasing from 0 to 0.45 W.

Supplementary Video S3: High-speed optomechanical manipulation of an oil droplet along a MNF, with CW waveguided powers of 0.7 W and 2.2 W, respectively.

Supplementary Video S4: Optomechanical manipulation of an 8-μm-diameter silica microsphere via an evanescent field of a 410-nm-diameter MNF.

References

1. Xiao, L. M. & Birks, T. A. High finesse microfiber knot resonators made from double-ended tapered fibers. *Opt. Lett.* 36, 1098–1100 (2011).
2. Wu, Y., Rao, Y. J., Chen, Y. H. & Gong, Y. Miniature fiber-optic temperature sensors based on silica/polymer microfiber knot resonators. *Opt. Express* 17, 18142–18147 (2009).
3. Yuan, L. B. Effect of temperature and strain on fiber optic refractive index. *Acta Opt. Sin.* 17, 1713–1717 (1997).
4. Chen, H. et al. Fiber-optic temperature sensor interrogation technique based on an optoelectronic oscillator. *Opt. Eng.* 55, 031107 (2016).
5. Weber, M. J. *Handbook of Optical Materials* (CRC Press, 2003).
6. Haynes, W. M. *CRC Handbook of Chemistry and Physics* (CRC Press, 2014).
7. Ruddell, S. K., Webb, K. E., Takahata, M., Kato, S. & Aoki, T. Ultra-low-loss nanofiber Fabry-Perot cavities optimized for cavity quantum electrodynamics. *Opt. Lett.* 45, 4875–4878 (2020).
8. Skuja, L. Optically active oxygen-deficiency-related centers in amorphous silicon dioxide. *J. Non-Cryst. Solids* 239, 16–48 (1998).
9. Kajihara, K., Skuja, L., Hirano, M. & Hosono, H. Role of mobile interstitial oxygen atoms in defect processes in oxides: interconversion between oxygen-associated defects in SiO_2_ glass. *Phys. Rev. Lett.* 92, 015504 (2004).
10. Skuja, L., Hirano, M., Hosono, H. & Kajihara, K. Defects in oxide glasses. *Phys. Status Solidi C* 2, 15–24 (2005).
11. Liao, F. et al. Enhancing monolayer photoluminescence on optical micro/nanofibers for low-threshold lasing. *Sci. Adv.* 5, eaax7398 (2019).
12. Cao, M. et al. Wavelength dependence of nanosecond laser induced surface damage in fused silica from 260 to 1550 nm. *J. Appl. Phys.* 123, 135105 (2018).
13. Novotny, L. & Hecht, B. *Principles of nano-optics* (Cambridge university press, 2012).
14. Tong, L. M., Lou, J. Y. & Mazur, E. Single-mode guiding properties of subwavelength-diameter silica and silicon wire waveguides. *Opt. Express* 12, 1025–1035 (2004).
15. Grubsky, V. & Savchenko, A. Glass micro-fibers for efficient third harmonic generation. *Opt. Express* 13, 6798–6806 (2005).
16. Lægsgaard, J. Theory of surface second-harmonic generation in silica nanowires. *J. Opt. Soc. Am. B* 27, 1317–1324 (2010).
17. Kang, Y. et al. Ultrahigh-precision diameter control of nanofiber using direct mode cutoff feedback. *IEEE Photon. Technol. Lett.* 32, 219–222 (2020).
18. Akimov, D. A. et al. Generation of a spectrally asymmetric third harmonic with unamplified 30-fs Cr:forsterite laser pulses in a tapered fiber. *Appl. Phys. B* 76, 515–519 (2003).
19. Grubsky, V. & Feinberg, J. Phase-matched third-harmonic UV generation using low-order modes in a glass micro-fiber. *Opt. Commun.* 274, 447–450 (2007).
20. Wiedemann, U. et al. Measurement of submicrometre diameters of tapered optical fibres using harmonic generation. *Opt. Express* 18, 7693–7704 (2010).
21. Coillet, A. & Grelu, P. Third-harmonic generation in optical microfibers: from silica experiments to highly nonlinear glass prospects. *Opt. Commun.* 285, 3493–3497 (2012).
22. Lee, T. et al. Broadband third harmonic generation in tapered silica fibres. *Opt. Express* 20, 8503–8511 (2012).
23. Gouveia, M. A. et al. Second harmonic generation and enhancement in microfibers and loop resonators. *Appl. Phys. Lett.* 102, 201120 (2013).
24. Jiang, X. J. et al. Enhanced UV third-harmonic generation in microfibers by controlling nonlinear phase modulations. *Opt. Lett.* 44, 4191–4194 (2019).
25. Yuan, J. H. et al. Generation of second-harmonics near ultraviolet wavelengths from femtosecond pump pulses. *IEEE Photon. Technol. Lett.* 28, 1719–1722 (2016).
26. Cavanna, A. et al. Hybrid photonic-crystal fiber for single-mode phase matched generation of third harmonic and photon triplets. *Optica* 3, 952–955 (2016).
27. Lin, A. X., Ryasnyanskiy, A. & Toulouse, J. Tunable third-harmonic generation in a solid-core tellurite glass fiber. *Opt. Lett.* 36, 3437–3439 (2011).
28. Cheng, T. L. et al. Tunable third-harmonic generation in a chalcogenide-tellurite hybrid optical fiber with high refractive index difference. *Opt. Lett.* 39, 1005–1007 (2014).
29. Cheng, T. L. et al. Highly efficient second-harmonic generation in a tellurite optical fiber. *Opt. Lett.* 44, 4686–4689 (2019).
